# Integrated PERSEVERE and endothelial biomarker risk model predicts death and persistent MODS in pediatric septic shock: a secondary analysis of a prospective observational study

**DOI:** 10.1186/s13054-022-04070-5

**Published:** 2022-07-11

**Authors:** Mihir R. Atreya, Natalie Z. Cvijanovich, Julie C. Fitzgerald, Scott L. Weiss, Michael T. Bigham, Parag N. Jain, Adam J. Schwarz, Riad Lutfi, Jeffrey Nowak, Geoffrey L. Allen, Neal J. Thomas, Jocelyn R. Grunwell, Torrey Baines, Michael Quasney, Bereketeab Haileselassie, Christopher J. Lindsell, Matthew N. Alder, Hector R. Wong

**Affiliations:** 1 Division of Critical Care Medicine, MLC2005, Cincinnati Children’s Hospital Medical Center, Cincinnati Children’s Research Foundation, 3333 Burnet Avenue, Cincinnati, OH 45229 USA; 2 Department of Pediatrics, University of Cincinnati College of Medicine, Cincinnati, OH 45267 USA; 3 UCSF Benioff Children’s Hospital Oakland, Oakland, CA 94609 USA; 4 Children’s Hospital of Philadelphia, Philadelphia, PA 19104 USA; 5 Akron Children’s Hospital, Akron, OH 44308 USA; 6 Texas Children’s Hospital and Baylor College of Medicine, Houston, TX 77030 USA; 7 Children’s Hospital of Orange County, Orange, CA 92868 USA; 8 Riley Hospital for Children, Indianapolis, IN 46202 USA; 9 Children’s Hospital and Clinics of Minnesota, Minneapolis, MN 55404 USA; 10 Children’s Mercy Hospital, Kansas City, MO 64108 USA; 11 Penn State Hershey Children’s Hospital, Hershey, PA 17033 USA; 12 Children’s Healthcare of Atlanta at Egleston, Atlanta, GA 30322 USA; 13 University of Florida Health Shands Children’s Hospital, Gainesville, FL 32610 USA; 14 CS Mott Children’s Hospital at the University of Michigan, Ann Arbor, MI 48109 USA; 15 Lucile Packard Children’s Hospital Stanford, Palo Alto, CA 94304 USA; 16 Department of Biostatistics, Vanderbilt University Medical Center, Nashville, TN 37212 USA

**Keywords:** Sepsis, Septic shock, Multiple organ dysfunction syndrome, Endothelial dysfunction, Precision medicine, Biomarkers, Prognostic enrichment

## Abstract

**Background:**

Multiple organ dysfunction syndrome (MODS) is a critical driver of sepsis morbidity and mortality in children. Early identification of those at risk of death and persistent organ dysfunctions is necessary to enrich patients for future trials of sepsis therapeutics. Here, we sought to integrate endothelial and PERSEVERE biomarkers to estimate the composite risk of death or organ dysfunctions on day 7 of septic shock.

**Methods:**

We measured endothelial dysfunction markers from day 1 serum among those with existing PERSEVERE data. TreeNet® classification model was derived incorporating 22 clinical and biological variables to estimate risk. Based on relative variable importance, a simplified 6-biomarker model was developed thereafter.

**Results:**

Among 502 patients, 49 patients died before day 7 and 124 patients had persistence of MODS on day 7 of septic shock. Area under the receiver operator characteristic curve (AUROC) for the newly derived PERSEVEREnce model to predict death or day 7 MODS was 0.93 (0.91–0.95) with a summary AUROC of 0.80 (0.76–0.84) upon tenfold cross-validation. The simplified model, based on IL-8, HSP70, ICAM-1, Angpt2/Tie2, Angpt2/Angpt1, and Thrombomodulin, performed similarly. Interaction between variables—ICAM-1 with IL-8 and Thrombomodulin with Angpt2/Angpt1—contributed to the models’ predictive capabilities. Model performance varied when estimating risk of individual organ dysfunctions with AUROCS ranging from 0.91 to 0.97 and 0.68 to 0.89 in training and test sets, respectively.

**Conclusions:**

The newly derived PERSEVEREnce biomarker model reliably estimates risk of death or persistent organ dysfunctions on day 7 of septic shock. If validated, this tool can be used for prognostic enrichment in future pediatric trials of sepsis therapeutics.

**Graphical abstract:**

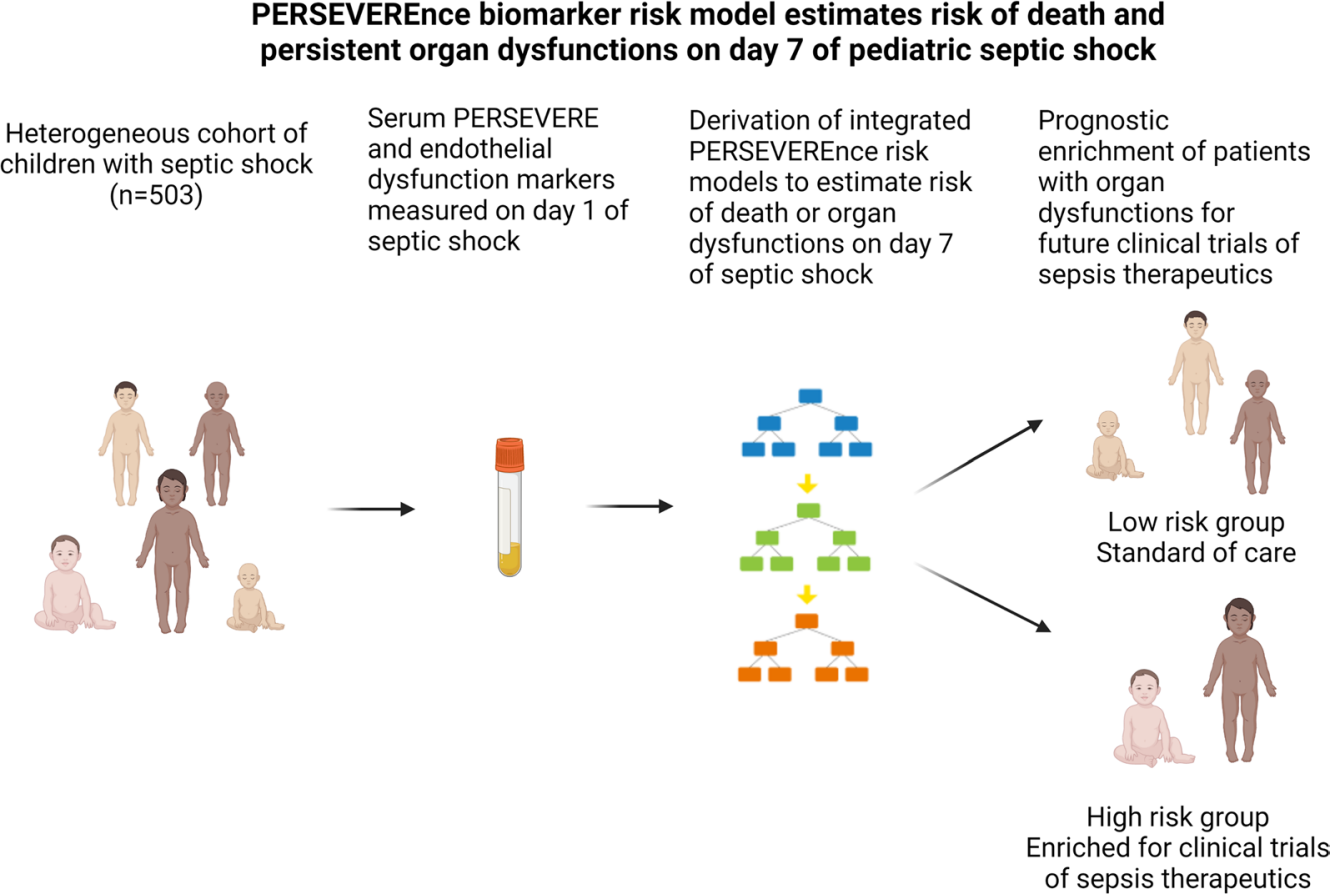

**Supplementary Information:**

The online version contains supplementary material available at 10.1186/s13054-022-04070-5.

## Introduction

Septic shock is a major problem among children admitted to pediatric intensive care units (PICU) [1]. Patients with persistent multiple organ dysfunction syndrome (MODS) are at highest risk of early [1] and late mortality [2], new medical device acquisition [3], and long-term neurocognitive impairment [4]. The current standard of care—antibiotics and intensive care [5]—although appropriate for most patients, may be insufficient for those with MODS. Early identification of patients who may benefit from timely institution of targeted sepsis therapeutics remains a challenge.

Clinical and biological heterogeneity among septic patients has long confounded efforts to develop efficacious therapeutics [6]. Precision medicine approaches offer potential solutions to sift through this underlying heterogeneity [7]. Prognostic enrichment through biomarker-based risk stratification may allow for identification of patients at high risk of death or persistent organ dysfunctions who can be targeted for enrollment in future clinical trials of sepsis therapeutics. Conversely, those deemed low risk can receive standard care and not be subject to potentially harmful therapies [8].

Organ dysfunctions in sepsis are partly driven by interaction of activated leukocytes with the endothelium, with subsequent dysregulation of cascades of inflammation and coagulation, and resultant tissue hypoperfusion [9, 10]. Despite this biological interplay, most studies of prognostic biomarkers in septic shock have considered the roles of these compartments separately rather than together. The serum Pediatric Sepsis Biomarker Risk Model (PERSEVERE), based on agnostic whole blood and leukocyte gene expression studies [11, 12], has been prospectively validated to estimate baseline risk of sepsis mortality [13–15]. More recently, it has been used to predict sepsis-associated acute kidney injury and myocardial dysfunction [16, 17], and pediatric acute respiratory distress syndrome [18]. In parallel, markers of endothelial dysfunction have been variably correlated with mortality and organ dysfunctions in adult [19] and pediatric sepsis [20]. The prognostic capabilities of the latter to determine clinical outcomes are yet to be validated.

Accordingly, we sought to determine whether integration of markers of endothelial dysfunction and PERSEVERE biomarkers measured on day 1 could reliably estimate risk of death or persistent organ dysfunctions on day 7 of septic shock in a large pediatric cohort.

## Methods

### Study design and patient selection

The study protocol was approved by Institutional Review Boards of participating institutions [13, 15]. Briefly, patients under the age of 18 years were recruited from multiple pediatric ICUs (PICU) across the USA between 2003 and 2019. Inclusion criteria were pediatric-specific consensus criteria for septic shock [21] and patients with existing PERSEVERE biomarker data. There were no study-related interventions except for blood draws. Clinical and laboratory data were available between day 1 through 7. Patients were followed for 28 days or until death, whichever came first. Organ dysfunctions were determined based on modifications to consensus criteria [21] and are detailed in Additional file 1. The primary outcome of interest was a composite that included patients who died before day 7 or those with ≥ 2 organ dysfunctions on day 7 of septic shock. We chose this composite outcome based on the assumption that (1) non-survivors died due to or with MODS, and that (2) non-survivors or those with persistence of organ dysfunctions on day 7, despite intensive organ support, represent a subset of patients with a yet unknown biological predilection that is potentially amenable to therapeutic intervention. Accordingly, there is sufficient clinical equipoise within this collective of patients to justify efforts for enrichment in future clinical trials of novel or repurposed sepsis therapeutics. Secondary outcomes were day 7 cardiovascular, respiratory, renal, hepatic, hematologic, and neurologic dysfunction.

### PERSEVERE biomarkers

Concentrations of interleukin 8 (IL-8), heat shock protein 70 kDA (HSP70), C–C chemokine ligand 3 (CCL3), C–C chemokine ligand 4 (CCL4), granzyme B (GZMB), interleukin 1 α (IL-1a), and matrix metallopeptidase 8 (MMP8) were previously measured in day 1 serum [13, 15].

### Endothelial dysfunction markers

Concentrations of Intercellular adhesion molecule-1 (ICAM-1), Thrombomodulin, Angiopoietin-1 (Angpt-1), Angiopoietin-2 (Angpt-2), Tyrosine kinase with immunoglobulin-like loops and epidermal growth factor homology domains-2 (Tie-2), Vascular cell adhesion molecule-1 (VCAM-1), P-selectin, E-selectin, Platelet, and endothelial cell adhesion molecule-1 (PECAM-1) were measured in day 1 serum by Luminex assays (R&D Systems, MN), according to the manufacturer’s specifications.

### Statistical analyses and modeling

Statistical analyses were performed with Minitab**®** software (v21.1, PA). Demographic and clinical data were summarized with percentages or median with interquartile ranges. Differences between groups were determined by chi-square test for categorical variables and by one-way analysis of variance (ANOVA) for continuous variables. A p value of 0.05 was used to test statistical significance. Twenty-two predictors including clinical age, serum lactate, PRISM III score [22], and day 1 vasoactive inotropic score (VIS) [23] and biological variables (ratios of Angpt-2/Tie-2 and Angpt-2/Angpt-1 and Log (10)-transformed PERSEVERE and endothelial marker concentrations) were considered. Multiple logistic regression was used to test the association between individual biomarkers and risk of primary outcome of interest after adjusting for age, sex, and PRISM III score.

Predictive analytics module with automated machine learning tool was used to discover the best model among TreeNet®, Random Forests®, Classification and Regression Tree (CART®), and logistic regression models. TreeNet® consistently provided the least misclassification and was chosen for model derivation. TreeNet® models, which rely on stochastic gradient boosting, consist of several hundred CART® trees with a limited number of terminal nodes. Iterative steps using recursive data sampling are used to grow additional trees to explain residual error from previous trees. While CART® classification only captures interactions of predictor variables in very specific combinations that influence the outcome together, TreeNet® allows for the capture of the overall effect of one predictor variable over another.

Models were weighted to ensure equal sample size across classes to overcome unequal distribution of classes of organ dysfunctions in the training dataset. Second-order interactions between biological variables were allowed. An event probability threshold of 0.45 was used to optimize model sensitivity. Tenfold cross-validation was used in test sets. Relative variable importance, defined as percent improvement with respect to the top predictor, was used to select variables to develop a simplified model. Test characteristics of risk prediction models including area under the receiver operator characteristic curve (AUROC), positive and negative predictive values, and likelihood ratios were determined. Percent of total squared error and % squared error for the top two-way interactions between biological variables were assessed. Finally, given the black box nature of TreeNet® models, alternative CART® model was derived to promote open science and allow for external validation. Briefly, models were weighted to match sample frequencies and minimum misclassification cost was chosen to select the optimal tree. Class probability method and tenfold cross-validation were used. CART® trees were pruned to ensure that terminal nodes had > 5% of patients of the root node.

## Results

### Baseline characteristics

A total of 502 patients with PERSEVERE and endothelial marker data were included in this study. Table 1 shows the demographic data of the cohort by the presence of death or day 7 MODS. Over one-third of the cohort (*n* = 173, 34.5%) patients had the primary outcome of interest, including 49 patients who died within the first 7 days of septic shock. Patients with death or day 7 MODS had higher day 1 VIS scores, were more likely to have received steroids, required more organ support, and had fewer ICU free days. Interrelationship between individual organ dysfunctions is detailed in Additional File 1. Concentrations of individual biomarkers by primary outcome and multiple logistic regression analyses to test their association with death or day 7 MODS, adjusted for age, sex, and PRISM III score, are detailed in Additional File 2.

**Table 1:** Demographic characteristics and clinical outcomes according to death or day 7 MODS in pediatric septic shock

Variable	No death or day 7 MODS	Death or day 7 MODS	*P* value
*n* (%)	329 (65.5%)	173 (34.5%)	
Age*	4.2 (1.5, 8.2)	2.7 (0.9, 6.6)	0.007
Sex, M, *n* (%)	170 (51.6%)	92 (53.2%)	0.748
PRISM III**	10 (6, 15)	15 (9)	< 0.001
Day 1 VIS	10 (2, 21)	20 (5, 50)	< 0.001
Lactate	1.2 (0, 2.3)	1.9 (0.9, 5.1)	< 0.001
Positive blood culture	62 (18.8%)	44 (25.4%)	0.086
Positive culture (any)	174 (52.8%)	108 (62.4%)	0.016
Source of infection:			0.030
Pulmonary	64 (19.4%)	45 (26.0%)	
Extrapulmonary	110 (33.4%)	63 (36.4%)	
Organism			0.052
Gram positive	78 (23.7%)	44 (25.4%)	
Gram negative	60 (18.2%)	41 (23.6%)	
Viral	22 (6.6%)	13 (7.5%)	
Fungal	8 (2.4%)	3 (1.7%)	
**Organ dysfunctions on day 7**			
Max. number of organ failures	0 (0, 1)	2 (1, 3)	< 0.001
Cardiovascular	9	113	< 0.001
Respiratory	38	155	< 0.001
Renal	14	129	< 0.001
Hepatic	6	77	< 0.001
Hematologic	11	96	< 0.001
Neurologic	0	41	< 0.001
**Organ support on day 7**			
Vasoactive support	11 (3.3%)	103 (59.5%)	< 0.001
Mechanical ventilation	46 (13.9%)	158 (91.3%)	< 0.001
Renal replacement therapy	5 (1.5%)	66 (38.5%)	< 0.001
Steroids	158 (48.1%)	101 (58.3%)	0.027
28-day Mortality	2 (0.6%)	61 (35.3%)	< 0.001
PICU LOS days	5 (7)	12 (13)	< 0.001
PICU-free days	23 (7)	15 (16)	< 0.001

### Estimating the risk of death or persistent organ dysfunctions on day 7 of septic shock

**Fig. 1:**
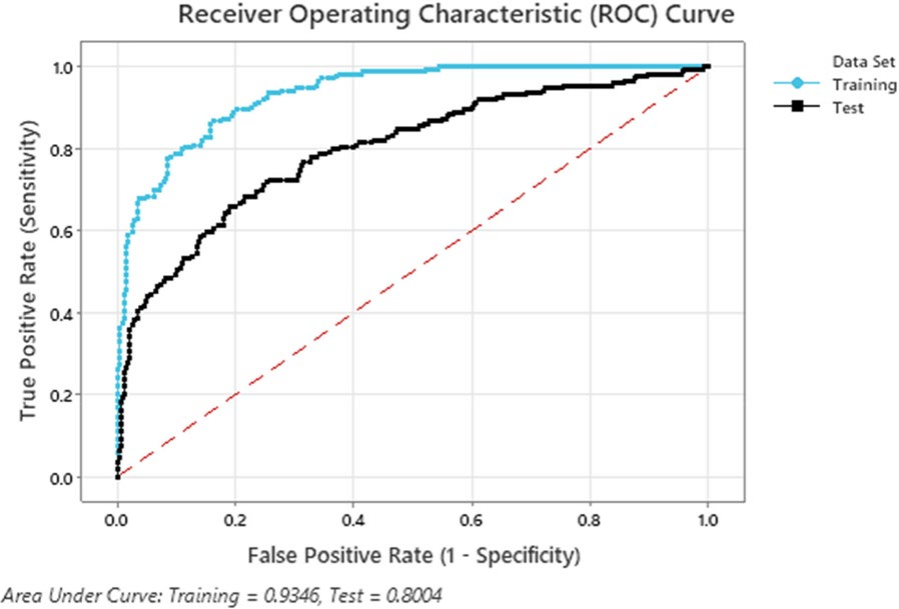
Area under the receiver operating characteristic (AUROC) curve for the 22-variable TreeNet® PERSEVEREnce model to estimate risk of death or day 7 MODS in children with septic shock

**Fig. 2:**
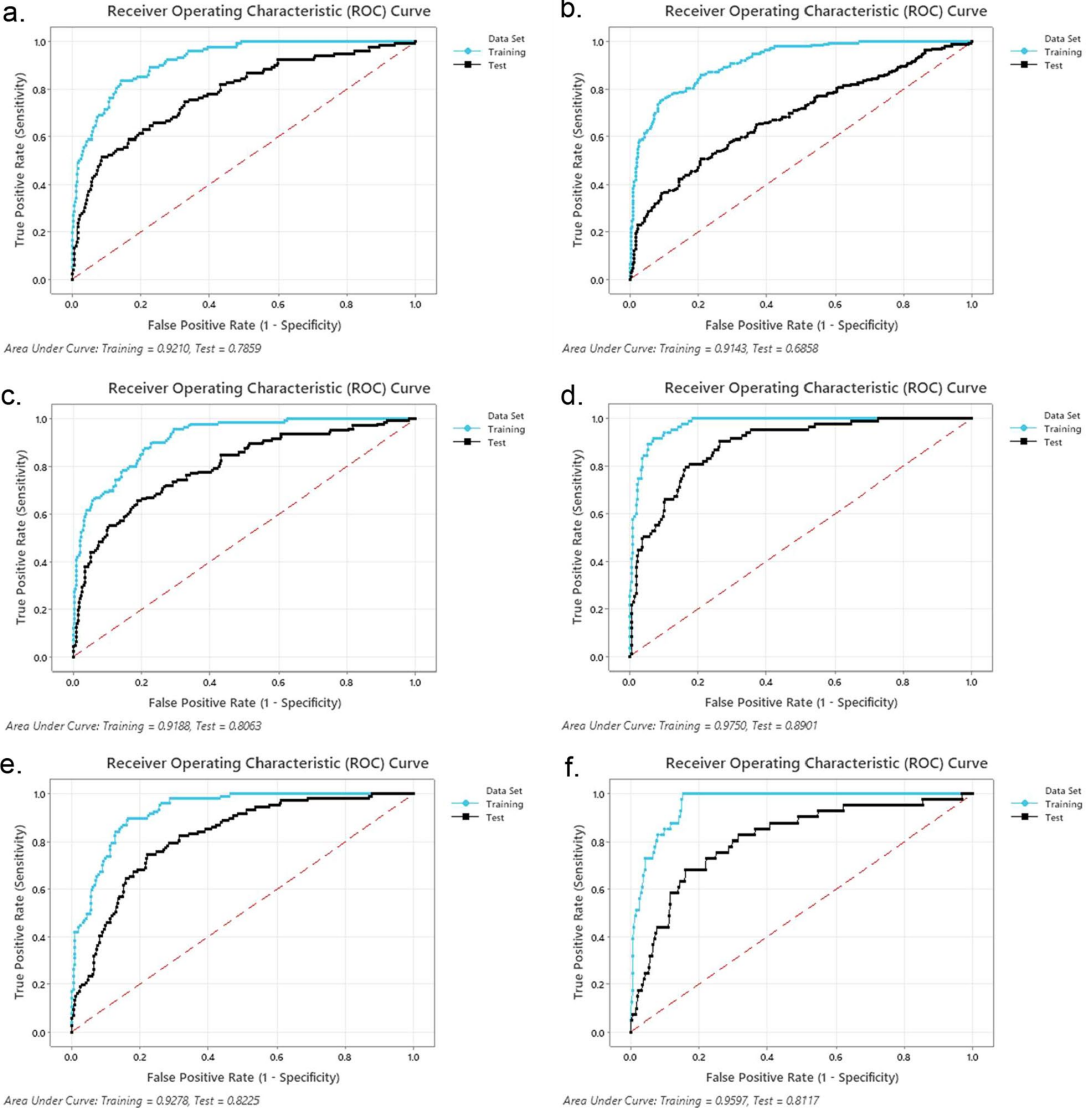
Area under the receiver operating characteristic (AUROC) curve for the 22-variable TreeNet® organ-specific PERSEVEREnce models to estimate risk of persistent **a** cardiovascular, **b** respiratory, **c** renal, **d**, hepatic, **e** hematologic, and **f** neurologic dysfunction on day 7 of pediatric septic shock

All 22 predictor variables were deemed important in the TreeNet® classification model. Three hundred trees were grown, and 220 was considered as the optimal number of trees. Table 2 shows test characteristics of the newly derived PERSEVEREnce model to estimate risk of death or day 7 MODS. The area under the receiver operator characteristic curve (AUROC) of the training set was 0.93 (95% CI 0.91–0.95) and 0.80 (95% CI 0.76–0.84) upon tenfold cross-validation as shown in Fig. 1. In comparison, summary AUROCs for clinical, PERSEVERE, and endothelial markers considered separately were 0.69 (95% CI 0.64–0.74), 0.73 (95% CI 0.68–0.78), and 0.75 (95% CI 0.71–0.79), respectively. The weighted misclassification rate of the PERSEVEREnce model was 0.16 and 0.27, and the negative predictive values were 92.1% (95% CI 88.3–94.9) and 83.7% (95% CI 78.8–87.6) in training and test sets, respectively. Relative variable importance of predictor variables and partial dependence plots of individual predictor variables are shown in Additional File 3. To test whether early deaths due to septic shock (≤ 48 h) skewed model performance, we conducted a sensitivity analysis with exclusion of these patients (*n* = 27); model performance was unchanged (data not shown). The test characteristics of models to estimate risk of individual organ dysfunctions are shown in Additional File 4. The AUROCs of organ-specific PERSEVEREnce risk models to predict cardiovascular, respiratory, renal, hepatic, hematologic, and neurologic dysfunctions on day 7 of septic shock ranged from 0.91 to 0.96 and 0.68 to 0.89 in test and training sets, respectively, and are shown in Fig. 2. The relative importance of predictor variables varied by organ dysfunction, as shown in Additional File 5.

**Table 2:** Test characteristics of 22-variable TreeNet® PERSEVEREnce model to estimate risk of death or day 7 MODS in children with septic shock

	Training set	Test set
AUROC	0.93 (0.91–0.95)	0.80 (0.76–0.84)
Weighted misclassification rate	0.16	0.26
True positive, *n*	150	125
False negative, *n*	23	48
False positive, *n*	58	83
True negative, *n*	271	246
Sensitivity %	86.7 (80.5, 91.2)	72.2 (64.8, 78.6)
Specificity %	82.3 (77.7, 86.2)	74.8 (69.7, 79.3)
Positive predictive value %	72.1 (65.4, 77.9)	60.1 (53.1, 66.7)
Negative predictive value %	92.1 (88.3, 94.9)	83.7 (78.8, 87.6)
Positive likelihood ratio	4.9 (3.9, 6.3)	2.9 (2.3, 3.5)
Negative likelihood ratio	0.2 (0.1, 0.3)	0.4 (0.3, 0.5)

Interaction between PERSEVERE and endothelial dysfunction markers consistently contributed to the prognostic capabilities of the PERSEVEREnce model to determine the risk of death or day 7 MODS. Interaction between ICAM-1 with IL-8 and thrombomodulin with Angpt-2/Angpt-1 accounted for 7.5 and 2.5% of total squared error and 13.2 and 10.3% of squared error, respectively, when estimating risk of death or day 7 MODS. Surface and contour plots of fitted half log odds of death or day 7 MODS explained by the two-way interaction of these variables and the top two-way interactions between biological variables in organ-specific PERSEVEREnce risk models are detailed in Additional File 6. The top interacting variables and the contribution of their interactions to the predictive capabilities of the models varied by organ dysfunction.

### Simplified PERSEVEREnce risk models

The top 6 biological variables including two PERSEVERE biomarkers IL-8 and HSP70 and four endothelial dysfunction markers ICAM-1, thrombomodulin, Angpt-2/Angpt-1, Angpt-2/Tie-2, selected based on a relative variable importance threshold of > 50% of top predictor, were used to develop simplified TreeNet® models to estimate risk of death or persistent organ dysfunctions on day 7 of septic shock. When estimating risk of death or day 7 MODS, 206 trees were considered as the optimal number of trees. The simplified PERSEVEREnce biomarker model had an AUROC of 0.89 (0.87–0.92) and 0.78 (0.75–0.83). The weighted misclassification rate was 0.18 and 0.27 in training and test sets, respectively. The test characteristics of the simplified model are detailed in Additional File 7. The AUROCs of the simplified organ-specific PERSEVEREnce models to predict cardiovascular, respiratory, renal, hepatic, hematologic, and neurologic dysfunctions on day 7 of septic shock ranged from 0.84 to 0.97 and 0.66 to 0.88 in training and test sets, respectively. The corresponding test characteristics are presented in Additional File 8. The top two-way interaction between the 6 variables and their contribution to each risk prediction model are shown in Additional File 9.

We derived a 7-terminal node PERSEVEREnce CART® model to estimate risk of death or day 7 MODS as shown in Additional File 10. Consistent with TreeNet® models, ICAM-1, IL-8, Angpt-2/Angpt-1, and Thrombomodulin influenced classification of patients and featured high in the tree. There were 1 low-risk terminal node (TN-1, with 10.6% risk), 3 intermediate risk terminal nodes (TN-2, 3, and 5, with 32.7–52.6% risk), and 3 high-risk terminal nodes (TN-4, 6, 7, with 60.7–89.3% risk of death or day 7 MODS). Of note, CART® models had significantly higher misclassification −0.65 and 0.84 in training and test sets—in comparison with TreeNet® models. The AUROC for CART® PERSEVEREnce model was 0.83 and 0.73 on tenfold cross-validation (data not shown).

### Prognostic enrichment of organ dysfunctions using PERSEVEREnce risk models

We tested whether simplified TreeNet® PERSEVEREnce models could result in meaningful enrichment of patients with death or persistent organ dysfunctions on day 7 of septic shock within the cohort. On reanalysis of the dataset, the PERSEVEREnce model correctly predicted those without death or day 7 MODS in 264 patients (true negatives) and incorrectly predicted the outcome of interest in 28 patients (false negatives). These true and false negative patients would be excluded from trials using PERSEVEREnce-based classification. In an enriched cohort, 69.4% of subjects would be expected to have death or day 7 MODS relative to 34.5% without enrichment. Using the PERSEVEREnce MODS model, the respective rates of cardiovascular, respiratory, renal, hepatic, hematologic, and neurologic dysfunctions in the enriched cohort, relative to one without enrichment, would be 47.6 vs 24.3%, 62.8 vs 38.4%, 53.8 vs 28.4%, 37.2 vs 16.5%, 43.8 vs 21.3%, and 17.6 vs 8.2%. The percent of patients requiring vasoactive support, mechanical ventilation, and renal replacement therapy in enriched vs. unenriched cohorts would be 44.3 vs 22.7%, 65.7 vs 40.7%, and 29.5 vs 14.2%, respectively. Organ-specific PERSEVEREnce models had comparable rates of enrichment for cardiovascular, respiratory, and renal dysfunction. However, enrichment to 59.2%, 59.1%, and 29.7% could be achieved for hepatic, hematologic, and neurologic dysfunctions, respectively.

## Discussion

We present newly derived PERSEVEREnce biomarker risk models to reliably estimate the risk of death or persistent organ dysfunctions on day 7 of septic shock in a large derivation cohort of pediatric septic shock patients. To the best of our knowledge, this is the first study to integrate whole blood/leukocyte and endothelial-derived biomarkers to predict sepsis-associated organ dysfunctions among children. Although 22 clinical and biological variables were considered during model development, we identified 6 variables, based on 7 serum biomarkers (IL-8, HSP70, ICAM-1, Thrombomodulin, Angpt-1, Angpt-2, and Tie-2), which contributed significantly to the predictive capabilities of the risk prediction models developed.

Interaction of IL-8 with ICAM-1 and thrombomodulin with Angpt-2/Angpt-1 contributed to the predictive capabilities of the PERSEVEREnce model to estimate risk of MODS. IL-8 and ICAM-1 are secreted by leukocytes and endothelial cells. They are involved in neutrophil adhesion, extravasation, and degranulation and contribute to endothelial dysfunction in sepsis [10, 24]. Thrombomodulin is expressed by endothelial cells and inhibits coagulation under normal circumstances [25]. An increase in Angiopoietin-2 relative to Angiopoietin-1, is thought to result in inhibition of Thrombomodulin and has been demonstrated to drive a procoagulant phenotype of endothelial cells [26]. Such recapitulation of key processes central to the pathophysiology of organ dysfunctions through agnostic machine learning algorithms lends biological plausibility to our risk prediction models. The relative importance of variables and their interactions varied in the organ-specific PERSEVEREnce models, which may reflect the unique interaction of activated leukocytes with the organotypic endothelium and highlights the complex biology of organ dysfunctions in sepsis.

PERSEVERE biomarkers will be used to retrospectively risk stratify patients and conduct secondary analyses of the randomized interventional trial of stress hydrocortisone in pediatric septic shock (SHIPPS, NCT03401398). Small-scale studies have demonstrated correlation between and feasibility of measuring PERSEVERE and endothelial markers among pediatric septic shock patients using MicroKine assays within 20 min [20]. If prospectively validated, it is conceivable that the PERSEVEREnce risk prediction models developed herein could be translated to enrich patients for adaptive trials of repurposed or novel sepsis therapeutics. Based on our data, a twofold enrichment in death or day 7 MODS, cardiovascular, respiratory, and renal dysfunctions could be achieved with the PERSEVEREnce biomarker model. The organ-specific PERSEVEREnce models could be expected to yield an over threefold enrichment for hepatic, hematologic, and neurological dysfunctions. We surmise that, because of the high rate of cardiovascular, respiratory, renal dysfunction, and interventions used to support these organ systems within the cohort, the respective organ-specific models did not yield further enrichment beyond that offered by the model used to estimate risk of death or day 7 MODS.

The evolution of organ dysfunctions is dynamic [27]. PERSEVEREnce models were developed based on day 1 biomarkers to predict death or persistent organ dysfunction on day 7 with a high negative predictive value (NPV). It is plausible that biomarkers measured later in the sepsis course could result in a temporal reclassification of risk. Further, artificial intelligence models based on clinical and laboratory data have recently shown promise in identifying patients at risk of MODS in critically ill children with a high positive predictive value [28]. As such these efforts may be viewed as complementary and, if prospectively validated, could be deployed either concurrently or sequentially to recalibrate risk of organ dysfunctions over time.

There are several limitations to our study. (1) 85% of patients had day 1 MODS in our cohort and only 14% patients had new and progressive MODS (NPMODS), the latter defined as those with single organ dysfunction on day 1 who go on to meet MODS criteria or those with day 1 MODS who accrue at least one additional organ dysfunction between day 1 and 7. In comparison, 58% of patients in the SPROUT study had day 1 MODS with 26% developing NPMODS [1]. We posit that PERSEVEREnce-based classification would perform equally well or better in a less critically ill cohort, given the relatively high negative predictive value of the model. (2) We did not consider phenotypes of pediatric sepsis MODS such as thrombocytopenia-associated multiorgan failure (TAMOF), immunoparalysis, and sequential MOF (SMOF) [29]. It is therefore likely that there is substantial heterogeneity within the subset of MODS patients within our cohort. Future studies may enable phenotype-specific risk prediction models. (3) Organ dysfunctions were based on modifications to consensus criteria established in 2005. Renal dysfunction was based on updated KDIGO criteria [30]. On the other hand, the definition of neurological dysfunction was restricted to those with Glasgow coma scale < 5, fixed dilated pupils, or the intracranial pressure > 20 mm Hg. The retrospective nature of the study limited our ability to consider contemporary features, such as neuromuscular weakness and delirium, and highlight the urgent need for revised criteria for organ dysfunctions in pediatric sepsis [31]. (4) PERSEVERE serum biomarkers were originally derived based on gene expression signatures correlated with 28-day mortality, not MODS, among children with septic shock. Efforts are currently underway to identify candidate biomarkers that are correlated specifically with the MODS signature. (5) The endothelial markers chosen for this study were based on their putative role in sepsis pathophysiology and previous association studies [19]. Therefore, it is plausible that other endothelial markers may further optimize model performance.

## Conclusions

In a large cohort of critically ill children, we integrated PERSEVERE and endothelial markers to reliably estimate risk of death or persistent organ dysfunctions on day 7 of septic shock. If prospectively validated, PERSEVEREnce biomarkers may facilitate prognostic enrichment of pediatric patients with organ dysfunctions in future pediatric trials of sepsis therapeutics.

## Supplementary Information

**Additional file 1**. Definitions and correlation of organ dysfunctions in the study.

**Additional file 2**. Univariate and multivariate associations between predictor variables and risk of death or day 7 MODS among children with septic shock.

**Additional file 3**. Relative variable importance and one predictor partial dependence plots of 22 predictor variables and risk of death or day 7 MODS among children with septic shock.

**Additional file 4**. Test characteristics of 22-variable organ-specific PERSEVEREnce risk models to estimate risk of death or day 7 MODS.

**Additional file 5**. Relative variable importance of predictors in the 22-variable organ-specific PERSEVEREnce risk models.

**Additional file 6**. Figure shows top two-way interaction between variables in PERSEVEREnce model to estimate risk of death or day 7 MODS. Table shows top two-way interactions in the organ-specific PERSEVEREnce models.

**Additional file 7**. AUROC (Figure) and test characteristics (Table) for simplified PERSEVEREnce model to estimate risk of death or day 7 MODS.

**Additional file 8**. Test characteristics of 6-variable organ-specific PERSEVEREnce risk models.

**Additional file 9**. Top two-way interactions in the simplified organ-specific PERSEVEREnce risk models.

**Additional file 10**. Seven terminal node CART tree model to estimate risk of death or day 7 MODS in children with septic shock.

## Data Availability

All data generated or analyzed during this study are included in this published article and its supplementary information files. The datasets used and/or analyzed during the current study are available from the corresponding author on reasonable request.

## References

[1] Weiss SL, Fitzgerald JC, Pappachan J (2015). Global epidemiology of pediatric severe sepsis: the sepsis prevalence, outcomes, and therapies study. Am J Respir Crit Care Med.

[2] Zimmerman JJ, Banks R, Berg RA (2020). Critical illness factors associated with long-term mortality and health related quality of life morbidity following community-acquired pediatric septic shock. Crit Care Med.

[3] Carlton EF, Donnelly JP, Hensley MK (2020). New medical device acquisition during pediatric severe sepsis hospitalizations. Crit Care Med.

[4] Iwashyna TJ, Ely EW, Smith DM (2010). Long-term cognitive impairment and functional disability among survivors of severe sepsis. JAMA.

[5] Weiss SL, Peters MJ, Alhazzani W (2020). Surviving sepsis campaign international guidelines for the management of septic shock and sepsis-associated organ dysfunction in children. Pediatr Crit Care Med.

[6] Marshall JC (2014). Why have clinical trials in sepsis failed?. Trends Mol Med.

[7] Atreya MR, Wong HR (2019). Precision medicine in pediatric sepsis. Curr Opin Pediatr.

[8] Stanski NL, Wong HR (2020). Prognostic and predictive enrichment in sepsis. Nat Rev Nephrol.

[9] Lelubre C, Vincent J-L (2018). Mechanisms and treatment of organ failure in sepsis. Nat Rev Nephrol.

[10] Joffre J, Hellman J, Ince C (2020). Endothelial responses in sepsis. Am J Respir Crit Care Med.

[11] Wong HR, Cvijanovich NZ, Allen GL (2011). Validation of a gene expression-based subclassification strategy for pediatric septic shock. Crit Care Med.

[12] Wong HR, Cvijanovich NZ, Anas N (2015). Developing a clinically feasible personalized medicine approach to pediatric septic shock. Am J Respir Crit Care Med.

[13] Wong HR, Salisbury S, Xiao Q (2012). The pediatric sepsis biomarker risk model. Crit Care.

[14] Wong HR, Cvijanovich NZ, Anas N (2016). PERSEVERE-II: redefining the pediatric sepsis biomarker risk model with septic shock phenotype. Crit Care Med.

[15] Wong HR, Caldwell JT, Cvijanovich NZ *et al.* Prospective clinical testing and experimental validation of the Pediatric Sepsis Biomarker Risk Model. *Sci Transl Med.* 2019;**11**, doi:10.1126/scitranslmed.aax9000. 10.1126/scitranslmed.aax9000 PMC7720682 31723040

[16] Stanski NL, Stenson EK, Cvijanovich NZ (2020). PERSEVERE biomarkers predict severe acute kidney injury and renal recovery in pediatric septic shock. Am J Respir Crit Care Med.

[17] Lautz AJ, Wong HR, Ryan TD (2021). Pediatric sepsis biomarker risk model biomarkers and estimation of myocardial dysfunction in pediatric septic shock. Pediatr Crit Care Med.

[18] Yehya N, Wong HR (2018). Adaptation of a biomarker-based sepsis mortality risk stratification tool for pediatric acute respiratory distress syndrome. Crit Care Med.

[19] Xing K, Murthy S, Liles WC (2012). Clinical utility of biomarkers of endothelial activation in sepsis-a systematic review. Crit Care.

[20] Carlton EF, McHugh WM, McDonough K (2020). Markers of endothelial dysfunction and cytokines in high-risk pediatric patients with severe sepsis. Am J Respir Crit Care Med.

[21] Goldstein B, Giroir B, Randolph A (2005). International pediatric sepsis consensus conference: definitions for sepsis and organ dysfunction in pediatrics. Pediatr Crit Care Med.

[22] Pollack MM, Patel KM, Ruttimann UE (1997). The pediatric risk of mortality III–acute physiology score (PRISM III-APS): a method of assessing physiologic instability for pediatric intensive care unit patients. J Pediatr.

[23] McIntosh AM, Tong S, Deakyne SJ (2017). Validation of the vasoactive-inotropic score in pediatric sepsis*. Pediatr Crit Care Med.

[24] Le KTT, Chu X, Jaeger M *et al.* Leukocyte-released mediators in response to both bacterial and fungal infections trigger IFN pathways, independent of IL-1 and TNF-α, in endothelial cells. *Front Immunol. *2019;**10**. 10.3389/fimmu.2019.02508 PMC6824321 31708927

[25] Giri H, Panicker SR, Cai X (2021). Thrombomodulin is essential for maintaining quiescence in vascular endothelial cells. Proc Natl Acad Sci.

[26] Daly C, Qian X, Castanaro C (2018). Angiopoietins bind thrombomodulin and inhibit its function as a thrombin cofactor. Sci Rep.

[27] Marshall JC, Deutschman CS (2021). The multiple organ dysfunction syndrome: syndrome, metaphor, and unsolved clinical challenge. Crit Care Med.

[28] Bose SN, Greenstein JL, Fackler JC (2021). Early prediction of multiple organ dysfunction in the pediatric intensive care unit. Front Pediatr.

[29] Carcillo JA, Podd B, Aneja R (2017). Pathophysiology of pediatric multiple organ dysfunction syndrome. Pediatr Crit Care Med.

[30] Kellum JA, Lameire N, Aspelin P *et al.* Kidney disease: improving global outcomes (KDIGO) acute kidney injury work group. KDIGO clinical practice guideline for acute kidney injury. *Kidney Int Suppl* 2012;**2**:1–138.

[31] Schlapbach LJ, Weiss SL, Bembea MM (2022). Scoring systems for organ dysfunction and multiple organ dysfunction: the PODIUM consensus conference. Pediatrics.

