# Supplementary material for: Integrated PERSEVERE and endothelial biomarker risk model predicts death and persistent MODS in pediatric septic shock: a secondary analysis of a prospective observational study
Source: Crit Care. 2022 Jul 11;26:210. doi: 10.1186/s13054-022-04070-5 (PMC9275255; doi:10.1186/s13054-022-04070-5)
Supplement: Supplementary file 1 — Additional file 1. Definitions and correlation of organ dysfunctions in the study. [file 13054_2022_4070_MOESM1_ESM.pdf]

Additional File 1:

Definitions of organ dysfunctions:

Organ dysfunctions were determined based on modifications to pediatric consensus criteria[1]. We sought to account for pre-existing conditions and capture acute organ dysfunctions related to index septic shock admission. Accordingly, patients with pre-existing conditions were required to meet more stringent criteria for organ dysfunctions to be considered related to sepsis.

Cardiovascular dysfunction: Patients without pre-existing heart disease were considered to have cardiac dysfunction if had low mean arterial pressure for age, low heart rate for age, requirement of vasoactive support or cardiac arrest. Patients with pre-existing hx of congenital heart disease or pulmonary hypertension were only considered to have new cardiovascular dysfunction if they required vasoactive support or had cardiac arrest.

Respiratory dysfunction: Patients without pre-existing lung disease or pulmonary hypertension were considered to have respiratory dysfunction if meeting ≥ 1 of the following criteria: requiring endo-tracheal intubation for acute respiratory failure, mechanical ventilation for >24 hours, $PaO_2/FiO_2 < 250$, $PaCO_2 > 65$, $PaO_2 < 40$. Patients with pre-existing lung disease or pulmonary hypertension were considered to have respiratory dysfunction if meeting ≥ 2 of the above criteria.

Renal dysfunction: Patients with pre-existing renal disease were not considered to have acute renal dysfunction. Those without pre-existing renal disease, renal dysfunction was defined as meeting KDIGO [2] stage ≥ 2 acute kidney injury or need for

renal replacement therapy. Baseline creatinine was estimated by modified Schwarz or Pottel method, according to published methods [3].

Hepatic dysfunction: Patients with pre-existing hepatic disease were not considered to have acute hepatic dysfunction. Those without pre-existing disease, hepatic dysfunction was based on meeting at least one of the following 3 criteria: total bilirubin > 4 mg/dL, Alanine aminotransferase levels > 2 times upper limit for age and sex, and or gastrointestinal bleeding requiring greater than 20 ml/kg of blood transfusion.

Hematologic dysfunction: Patients without pre-existing hematologic disease, cancer, bone marrow transplantation were required to meet ≥ 1 of the following criteria international normalized ratio (INR) > 2, platelet count <80,000 per microliter of blood, or evidence of disseminated intravascular coagulopathy (DIC). Those with pre-existing conditions were required to meet ≥ 2 or the above criteria.

Neurologic dysfunction: Patients with pre-existing neurologic disease including those with hypoxic ischemic encephalopathy, cerebral palsy or epilepsy disorders, were not considered to have neurologic dysfunction. Among those without pre-existing conditions, patients had meet ≥ 1 of the following criteria: Glasgow coma scale < 5, fixed dilated pupils, or intracranial pressure > 20 millimeters of mercury to be considered to have neurologic dysfunction.

References:

1. Goldstein B, Giroir B, Randolph A *et al.* International pediatric sepsis consensus conference: definitions for sepsis and organ dysfunction in pediatrics. *Pediatr Crit Care Med* 2005;**6**:2–8.

2. Kellum JA, Lameire N, Aspelin P *et al.* Kidney disease: Improving global outcomes (KDIGO) acute kidney injury work group. KDIGO clinical practice guideline for acute kidney injury. *Kidney International Supplements* 2012;**2**:1–138.

3. Stanski NL, Stenson EK, Cvijanovich NZ *et al.* PERSEVERE Biomarkers Predict Severe Acute Kidney Injury and Renal Recovery in Pediatric Septic Shock. *Am J Respir Crit Care Med* 2020;**201**:848–55.

Supplemental Figure 1: a) Chord diagram represents inter-relationship between individual organ dysfunctions on day 7 of septic shock **b**) Correlogram between pairs of individual organ dysfunctions on day 7 of septic shock.

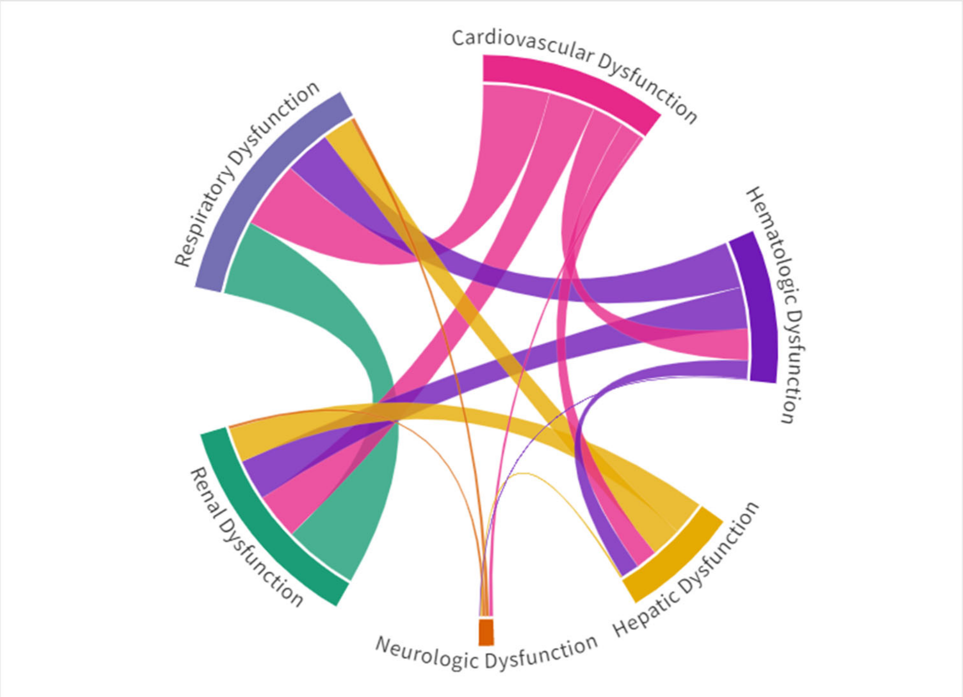

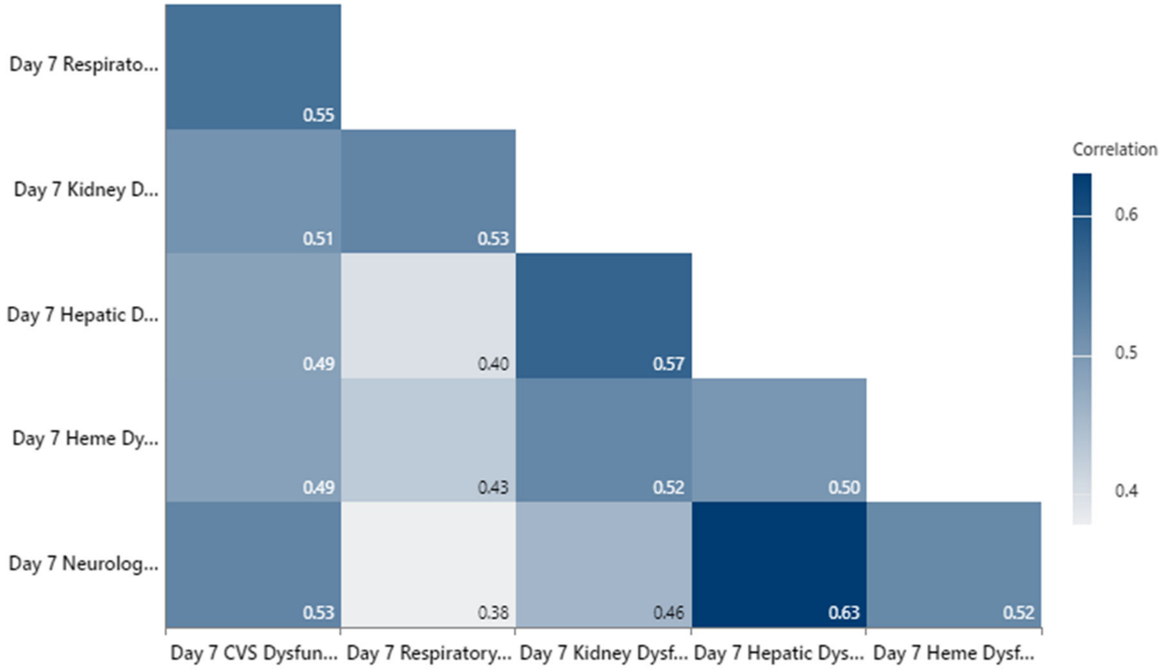

Supplemental Table 1: 28-day mortality among pediatric septic shock patients with and without day 7 organ dysfunctions.

	28-day mortality among patients without Day 7 Organ Dysfunction	28-day mortality among patients with Day 7 Organ Dysfunction	p value
Cardiovascular	9	54	<0.001
Respiratory	4	59	<0.001
Renal	5	58	<0.001
Hepatic	11	52	<0.001
Hematologic	5	58	<0.001
Neurologic	24	39	<0.001
