# Supplementary material for: Integrated PERSEVERE and endothelial biomarker risk model predicts death and persistent MODS in pediatric septic shock: a secondary analysis of a prospective observational study
Source: Crit Care. 2022 Jul 11;26:210. doi: 10.1186/s13054-022-04070-5 (PMC9275255; doi:10.1186/s13054-022-04070-5)
Supplement: Supplementary file 2 — Additional file 2. Univariate and multivariate associations between predictor variables and risk of death or day 7 MODS among children with septic shock. [file 13054_2022_4070_MOESM2_ESM.pdf]

Additional File 2:
Table 1. Concentrations of serum biomarkers according to death or day 7 MODS among children with septic shock.

Variable	No Day 7 MODS	Day 7 MODS	P value
**PERSEVERE biomarkers:**			
IL-8 (log10)	2.2 (1.8, 2.6)	2.9 (2.3, 3.9)	<0.001
HSP70 (log10)	5.8 (5.5, 6.0)	6.0 (5.7, 6.4)	<0.001
CXCL3 (log10)	1.9 (1.6, 2.0)	2.0 (1.7, 2.3)	<0.001
CXCL4 (log10)	2.0 (1.8, 2.3)	2.2 (1.9, 2.6)	<0.001
GZMB (log10)	1.1 (0.7, 1.5)	1.3 (0.8, 1.9)	0.002
IL-1α (log10)	0.2 (0, 0.8)	0.4 (0, 0.9)	0.081
MMP8 (log10)	4.6 (4.1, 5.0)	4.6 (4.1, 5.1)	0.549
**Endothelial biomarkers:**			
ICAM-1 (log10)	5.7 (5.6, 5.9)	5.9 (5.8, 6.1)	<0.001
Thrombomodulin (log10)	3.8 (3.7, 4.0)	4.0 (3.8, 4.2)	<0.001
Angpt1 (log10)	4.3 (4.0, 4.5)	4.0 (3.7, 4.3)	<0.001
Angpt2 (log10)	3.8 (3.6, 4.1)	4.1 (3.9, 4.4)	<0.001
Tie2 (log10)	4.4 (4.2, 4.5)	4.3 (4.1, 4.4)	<0.001
Angpt2/Angpt1	0.4 (0.2, 1.0)	1.4 (0.5, 3.5)	<0.001
Angpt2/Tie2	0.3 (0.2, 0.5)	0.8 (0.4, 1.4)	<0.001
VCAM-1 (log10)	6.4 (6.2, 6.7)	6.6 (6.3, 6.7)	0.001
P-selectin (log10)	4.8 (4.6, 5.0)	4.7 (4.6, 4.9)	0.002
E-selectin (log10)	4.9 (4.7, 5.1)	4.9 (4.6, 5.1)	0.374
PECAM-1 (log10)	4.3 (4.2, 4.5)	4.3 (4.3, 4.5)	0.607

Table 2. Multivariable logistic regression testing for the association between individual clinical and biological variables and death or day 7 MODS among children with septic shock.

Variable	Adjusted OR, 95% CI	P value
**Clinical variables:**		
Day 1 VIS	1.00 (1.00-1.01)	0.025
Lactate	1.14 (1.07-1.22)	<0.001
**PERSEVERE biomarkers:**		
IL-8 (log10)	2.70 (2.04-3.58)	<0.001
HSP70 (log10)	2.35 (1.60-3.45)	<0.001
CXCL3 (log10)	2.29 (1.41-3.69)	<0.001
CXCL4 (log10)	1.63 (1.06-2.51)	0.02
GZMB (log10)	1.40 (1.08-1.82)	0.01
IL-1α	1.21 (0.90-1.62)	0.20
MMP8	1.02 (0.80-1.32)	0.823
**Endothelial biomarkers:**		
ICAM-1 (log10)	23.29 (8.06-67.31)	<0.001
Thrombomodulin (log10)	19.73 (7.73-50.36)	<0.001
Angpt1 (log10)	0.31 (0.20-0.51)	<0.001
Angpt2 (log10)	6.84 (3.69-12.67)	<0.001
Tie2 (log10)	0.19 (0.07-0.51)	<0.001
Angpt2/Angpt1	1.16 (1.07-1.25)	<0.001
Angpt2/Tie2	3.13 (2.15-4.54)	<0.001
VCAM-1 (log10)	2.37 (1.21-4.62)	0.011
P-selectin (log10)	0.28 (0.12-0.66)	0.004
E-selectin (log10)	0.79 (0.44-1.41)	0.422
PECAM-1 (log10)	0.92 (0.37-2.28)	0.864

Adjusted for age, sex, and PRISM III *
