# Supplementary material for: Integrated PERSEVERE and endothelial biomarker risk model predicts death and persistent MODS in pediatric septic shock: a secondary analysis of a prospective observational study
Source: Crit Care. 2022 Jul 11;26:210. doi: 10.1186/s13054-022-04070-5 (PMC9275255; doi:10.1186/s13054-022-04070-5)
Supplement: Supplementary file 3 — Additional file 3. Relative variable importance and one predictor partial dependence plots of 22 predictor variables and risk of death or day 7 MODS among children with septic shock. [file 13054_2022_4070_MOESM3_ESM.pdf]

Supplemental Figure 2 a. Relative variable importance of 22 variable TreeNet Model to predict risk of death or day 7 MODS in pediatric septic shock

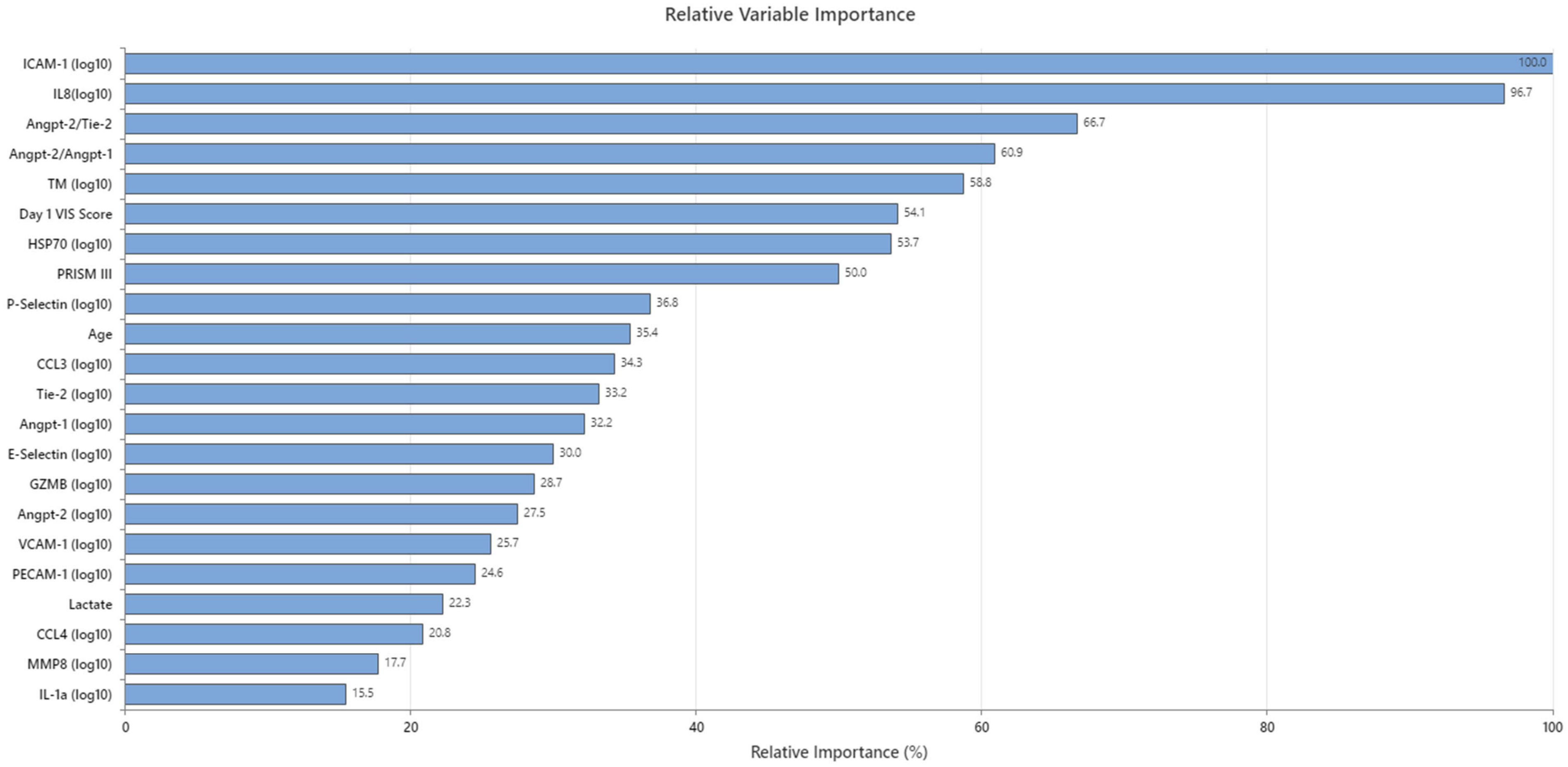

Variable importance measures model improvement when splits are made on a predictor. Relative importance is defined as % improvement with respect to the top predictor.

2b: One predictor partial dependence plots of clinical and biological variables

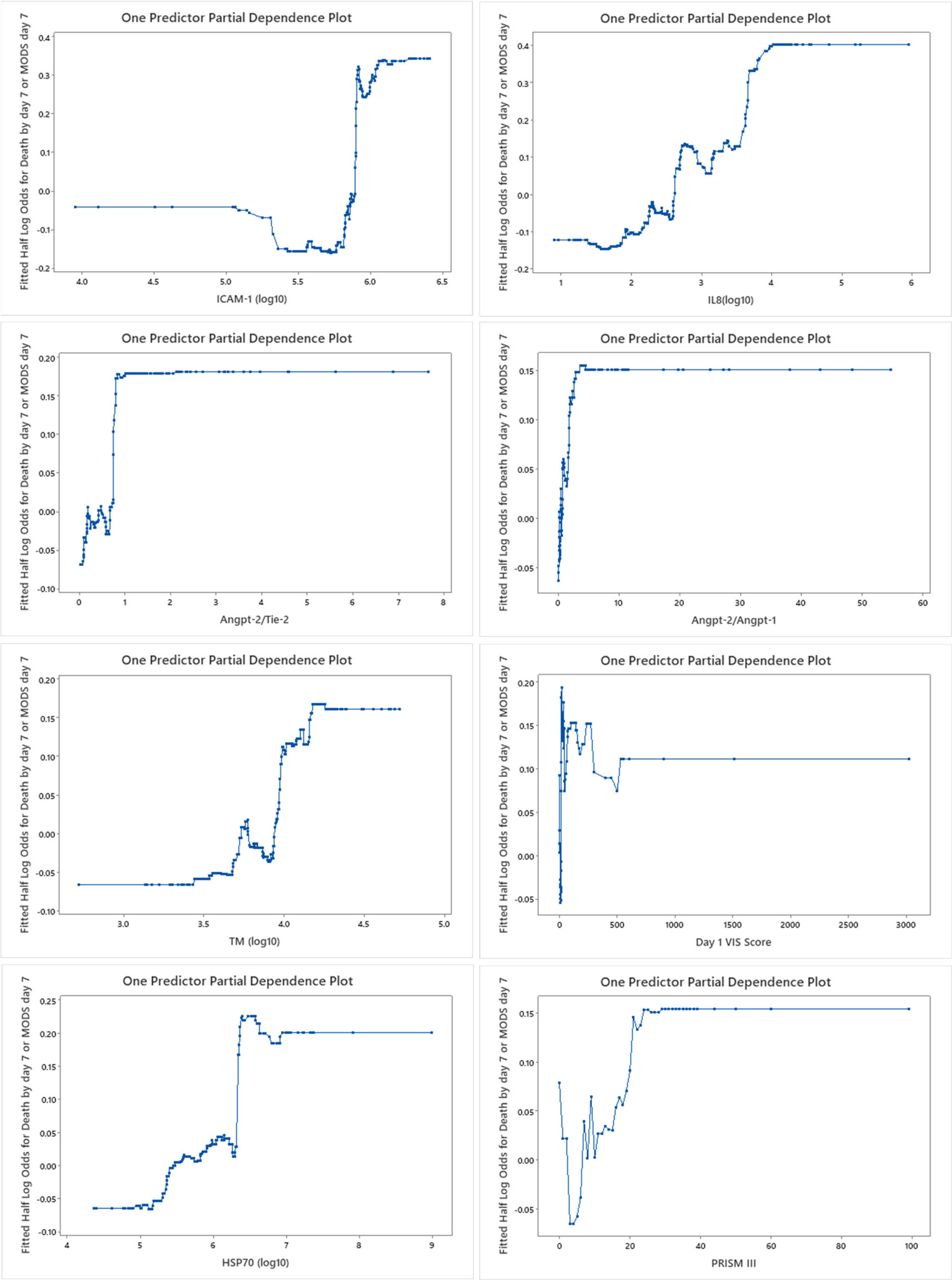

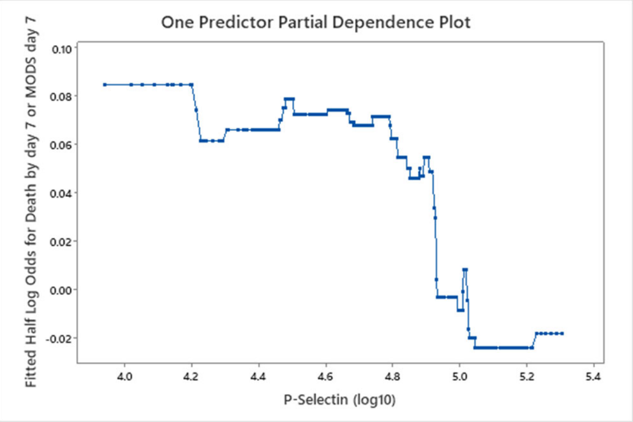
One Predictor Partial Dependence Plot
Fitted Half Log Odds for Death by day 7 or MODS day 7
0.10
0.08
0.06
0.04
0.02
0.00
-0.02
4.0
4.2
4.4
4.6
4.8
5.0
5.2
5.4
P-Selectin (log10)

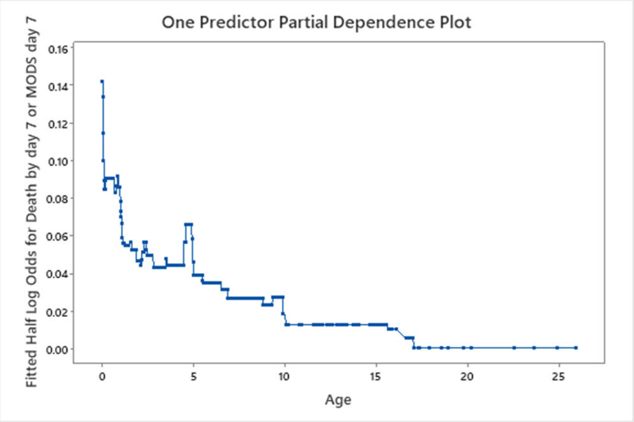
One Predictor Partial Dependence Plot
Fitted Half Log Odds for Death by day 7 or MODS day 7
0.16
0.14
0.12
0.10
0.08
0.06
0.04
0.02
0.00
0
5
10
15
20
25
Age

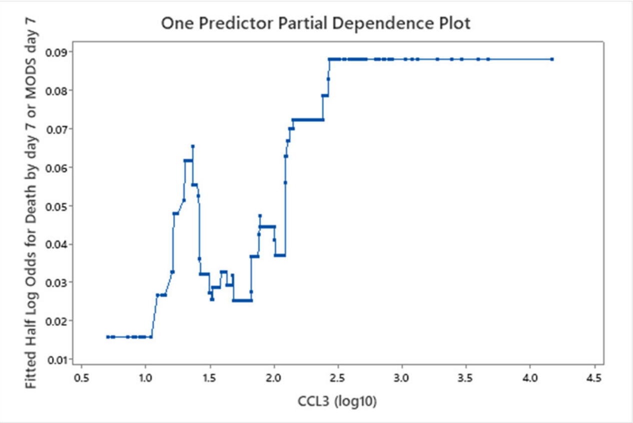
One Predictor Partial Dependence Plot
Fitted Half Log Odds for Death by day 7 or MODS day 7
0.09
0.08
0.07
0.06
0.05
0.04
0.03
0.02
0.01
0.5
1.0
1.5
2.0
2.5
3.0
3.5
4.0
4.5
CCL3 (log10)

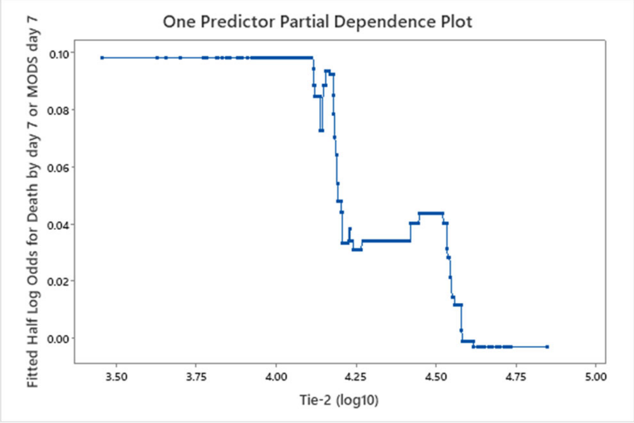
One Predictor Partial Dependence Plot
Fitted Half Log Odds for Death by day 7 or MODS day 7
0.10
0.08
0.06
0.04
0.02
0.00
3.50
3.75
4.00
4.25
4.50
4.75
5.00
Tie-2 (log10)

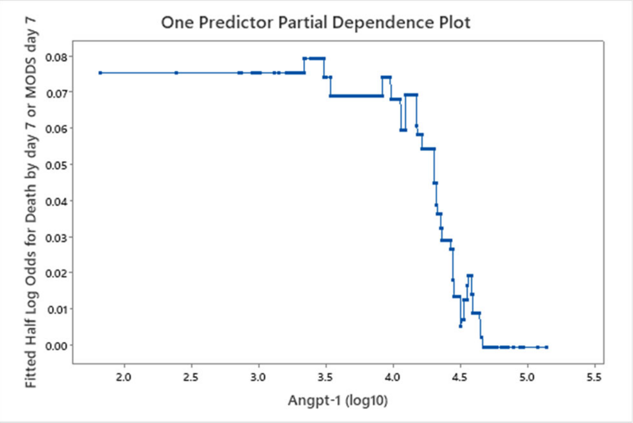
One Predictor Partial Dependence Plot
Fitted Half Log Odds for Death by day 7 or MODS day 7
0.08
0.07
0.06
0.05
0.04
0.03
0.02
0.01
0.00
2.0
2.5
3.0
3.5
4.0
4.5
5.0
5.5
Angpt-1 (log10)

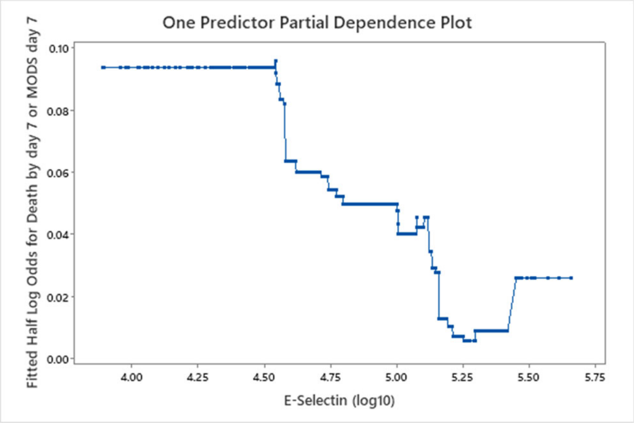
One Predictor Partial Dependence Plot
Fitted Half Log Odds for Death by day 7 or MODS day 7
0.10
0.08
0.06
0.04
0.02
0.00
4.00
4.25
4.50
4.75
5.00
5.25
5.50
5.75
E-Selectin (log10)

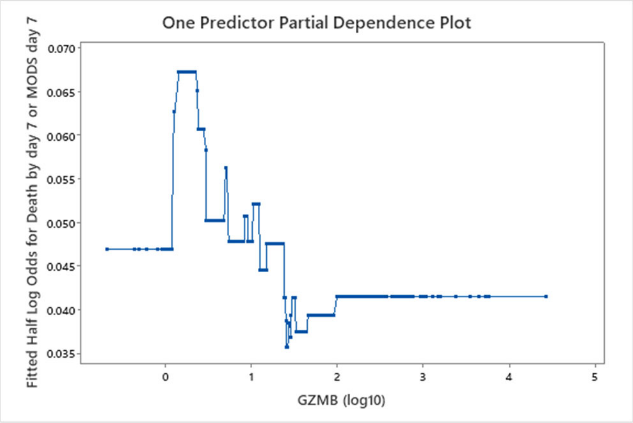
One Predictor Partial Dependence Plot
Fitted Half Log Odds for Death by day 7 or MODS day 7
0.070
0.065
0.060
0.055
0.050
0.045
0.040
0.035
0
1
2
3
4
5
GZMB (log10)

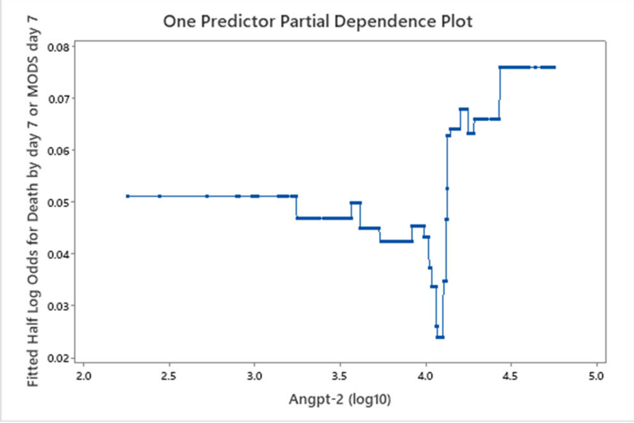
One Predictor Partial Dependence Plot
Fitted Half Log Odds for Death by day 7 or MODS day 7
0.08
0.07
0.06
0.05
0.04
0.03
0.02
2.0
2.5
3.0
3.5
4.0
4.5
5.0
Angpt-2 (log10)

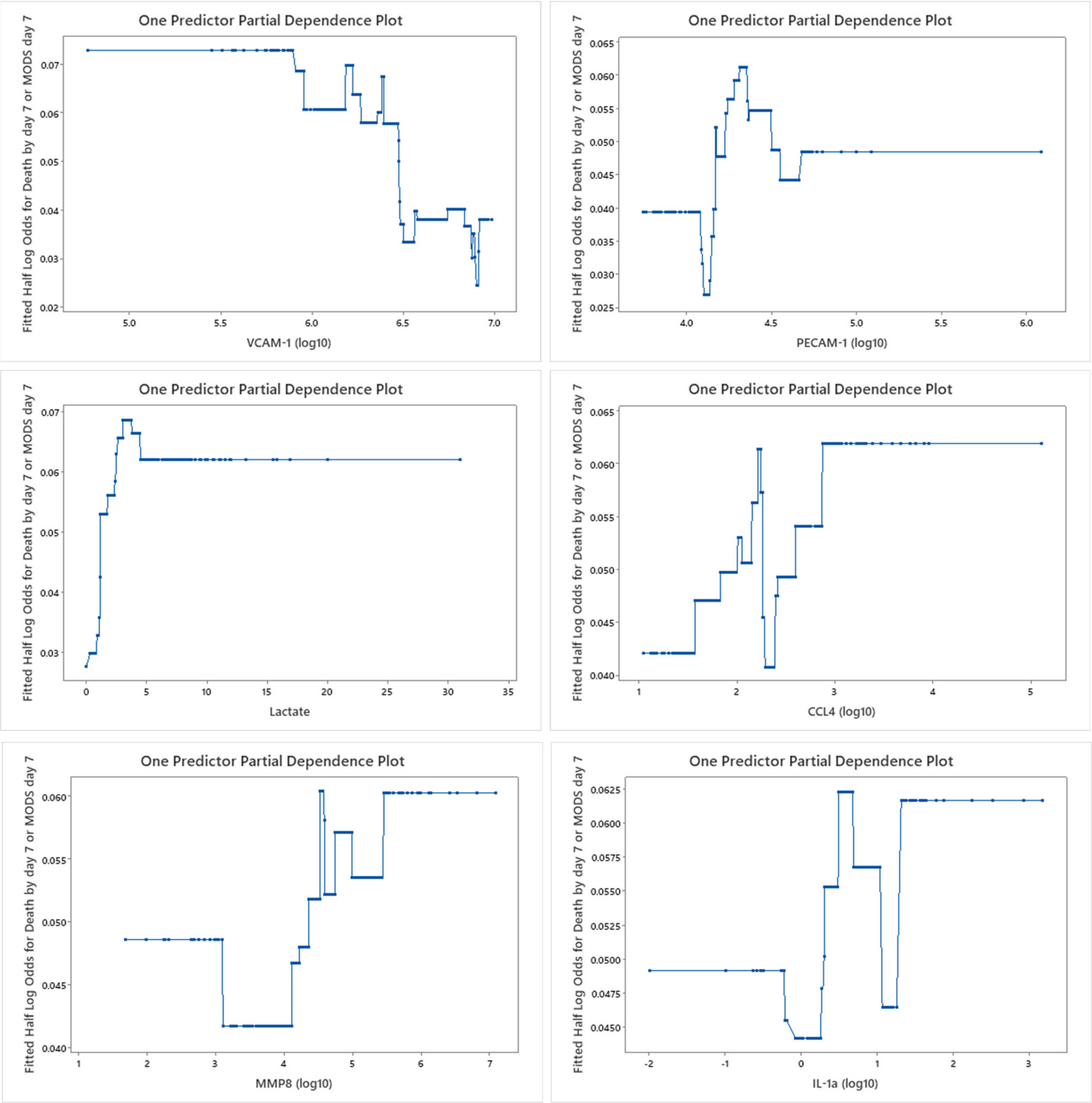
One Predictor Partial Dependence Plot
Fitted Half Log Odds for Death by day 7 or MODS day 7
0.07
0.06
0.05
0.04
0.03
0.02
5.0
5.5
6.0
6.5
7.0
VCAM-1 (log10)
One Predictor Partial Dependence Plot
Fitted Half Log Odds for Death by day 7 or MODS day 7
0.065
0.060
0.055
0.050
0.045
0.040
0.035
0.030
0.025
4.0
4.5
5.0
5.5
6.0
PECAM-1 (log10)
One Predictor Partial Dependence Plot
Fitted Half Log Odds for Death by day 7 or MODS day 7
0.07
0.06
0.05
0.04
0.03
0
5
10
15
20
25
30
35
Lactate
One Predictor Partial Dependence Plot
Fitted Half Log Odds for Death by day 7 or MODS day 7
0.065
0.060
0.055
0.050
0.045
0.040
1
2
3
4
5
CCL4 (log10)
One Predictor Partial Dependence Plot
Fitted Half Log Odds for Death by day 7 or MODS day 7
0.060
0.055
0.050
0.045
0.040
1
2
3
4
5
6
7
MMP8 (log10)
One Predictor Partial Dependence Plot
Fitted Half Log Odds for Death by day 7 or MODS day 7
0.0625
0.0600
0.0575
0.0550
0.0525
0.0500
0.0475
0.0450
-2
-1
0
1
2
3
IL-1a (log10)
