# Supplementary material for: Integrated PERSEVERE and endothelial biomarker risk model predicts death and persistent MODS in pediatric septic shock: a secondary analysis of a prospective observational study
Source: Crit Care. 2022 Jul 11;26:210. doi: 10.1186/s13054-022-04070-5 (PMC9275255; doi:10.1186/s13054-022-04070-5)
Supplement: Supplementary file 4 — Additional file 4. Test characteristics of 22-variable organ-specific PERSEVEREnce risk models to estimate risk of death or day 7 MODS. [file 13054_2022_4070_MOESM4_ESM.pdf]

**Additional file 4:**
Performance of 22 variable organ-specific TreeNet® PERSEVEREnce risk models.

| | | Training Set | Test Set |
|---|---|---|---|
| Day 7 Cardiovascular Dysfunction | | | |
| | AUROC | 0.92 (0.89, 0.94) | 0.79 (0.73, 0.84) |
| | Weighted misclassification rate | 0.17 | 0.29 |
| | True positive, n | 103 | 80 |
| | False negative, n | 19 | 42 |
| | False positive, n | 71 | 91 |
| | True negative, n | 309 | 289 |
| | Sensitivity % | 84.4 (76.4, 90.1) | 65.6 (56.3, 73.7) |
| | Specificity % | 81.3 (76.9, 85.0) | 76.1 (71.3, 80.2) |
| | Positive predictive value % | 59.1 (51.4, 66.4) | 46.7 (39.1, 54.5) |
| | Negative predictive value % | 94.2 (90.0, 96.3) | 87.3 (83.1, 90.6) |
| Day 7 Respiratory Dysfunction | | | |
| | AUROC | 0.91 (0.89, 0.94) | 0.68 (0.63, 0.73) |
| | Weighted misclassification rate | 0.17 | 0.36 |
| | True positive, n | 167 | 126 |
| | False negative, n | 26 | 67 |
| | False positive, n | 68 | 116 |
| | True negative, n | 241 | 193 |
| | Sensitivity % | 71.1 (64.7, 76.7) | 65.2 (58.1, 71.9) |
| | Specificity % | 77.9 (72.8, 82.4) | 62.4 (56.8, 67.8) |
| | Positive predictive value % | 71.1 (64.7, 76.6) | 52.1 (45.5, 58.4) |
| | Negative predictive value % | 78.0 (72.9, 82.4) | 74.2 (68.3, 79.3) |
| Day 7 Renal Dysfunction | | | |
| | AUROC | 0.91 (0.89, 0.94) | 0.80(0.76, 0.85) |
| | Weighted misclassification rate | 0.17 | 0.27 |
| | True positive, n | 123 | 103 |
| | False negative, n | 20 | 40 |
| | False positive, n | 73 | 96 |
| | True negative, n | 286 | 263 |
| | Sensitivity % | 86.1 (78.9, 91.1) | 72.1 (63.8, 77.7) |
| | Specificity % | 79.7 (75.1, 83.6) | 73.2 (68.3, 77.7) |
| | Positive predictive value % | 62.7 (55.5, 69.4) | 51.7 (44.6, 58.8) |
| | Negative predictive value % | 93.4 (89.9, 95.9) | 86.7 (82.3, 90.2) |

| | | Training Set | Test Set |
|---|---|---|---|
| Day 7 Hepatic Dysfunction | | | |
| | AUROC | 0.97 (0.96, 0.99) | 0.89 (0.85, 0.92) |
| | Weighted misclassification rate | 0.08 | 0.18 |
| | True positive, n | 79 | 66 |
| | False negative, n | 4 | 17 |
| | False positive, n | 53 | 68 |
| | True negative, n | 366 | 351 |
| | Sensitivity % | 95.1 (87.5, 98.4) | 79.5 (68.9, 87.2) |
| | Specificity % | 87.3 (83.6, 90.3) | 83.7 (79.8, 87.1) |
| | Positive predictive value % | 59.8 (50.9, 68.1) | 49.2 (40.5, 57.9) |
| | Negative predictive value % | 98.9 (97.1, 99.6) | 95.3 (92.5, 97.2) |
| Day 7 Hematologic Dysfunction | | | |
| | AUROC | 0.92 (0.90, 0.95) | 0.82 (0.78, 0.86) |
| | Weighted misclassification rate | 0.14 | 0.24 |
| | True positive, n | 96 | 79 |
| | False negative, n | 11 | 28 |
| | False positive, n | 75 | 87 |
| | True negative, n | 320 | 308 |
| | Sensitivity % | 89.7 (81.9, 94.5) | 73.8 (64.2, 81.6) |
| | Specificity % | 81.1 (76.7, 84.7) | 77.9 (73.4, 81.9) |
| | Positive predictive value % | 56.1 (48.3, 63.6) | 47.5 (39.8, 55.4) |
| | Negative predictive value % | 96.6 (93.9, 98.2) | 91.6 (88.1, 94.2) |
| Day 7 Neurologic Dysfunction | | | |
| | AUROC | 0.96 (0.94, 0.98) | 0.81 (0.74, 0.88) |
| | Weighted misclassification rate | 0.15 | 0.25 |
| | True positive, n | 38 | 28 |
| | False negative, n | 3 | 13 |
| | False positive, n | 68 | 73 |
| | True negative, n | 393 | 388 |
| | Sensitivity % | 92.7 (78.9, 98.1) | 68.2 (51.7, 81.4) |
| | Specificity % | 85.2 (81.6, 88.2) | 84.1 (80.4, 87.3) |
| | Positive predictive value % | 35.8 (26.9, 45.8) | 27.7 (19.5, 37.6) |
| | Negative predictive value % | 99.2 (97.6, 99.8) | 96.7 (94.3, 98.1) |
