# Supplementary material for: Integrated PERSEVERE and endothelial biomarker risk model predicts death and persistent MODS in pediatric septic shock: a secondary analysis of a prospective observational study
Source: Crit Care. 2022 Jul 11;26:210. doi: 10.1186/s13054-022-04070-5 (PMC9275255; doi:10.1186/s13054-022-04070-5)
Supplement: Supplementary file 5 — Additional file 5. Relative variable importance of predictors in the 22-variable organ-specific PERSEVEREnce risk models. [file 13054_2022_4070_MOESM5_ESM.pdf]

Supplemental Figure 3.
Relative variable importance of 22 variable TreeNet® PERSEVEREnce model to predict day 7 cardiovascular dysfunction.

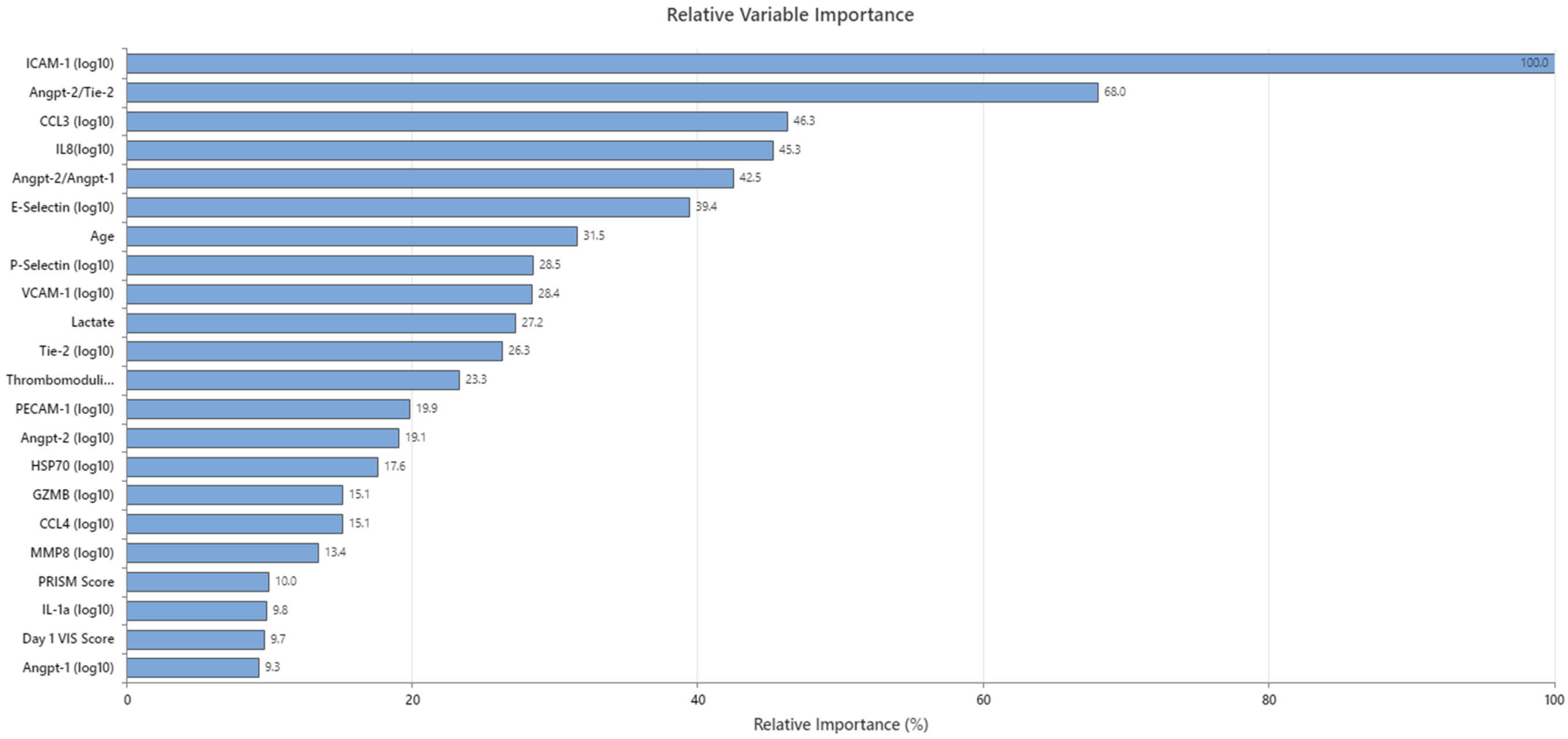

Variable importance measures model improvement when splits are made on a predictor. Relative importance is defined as % improvement with respect to the top predictor.

Relative variable importance of 22 variable TreeNet® PERSEVEREnce model to predict day 7 respiratory dysfunction

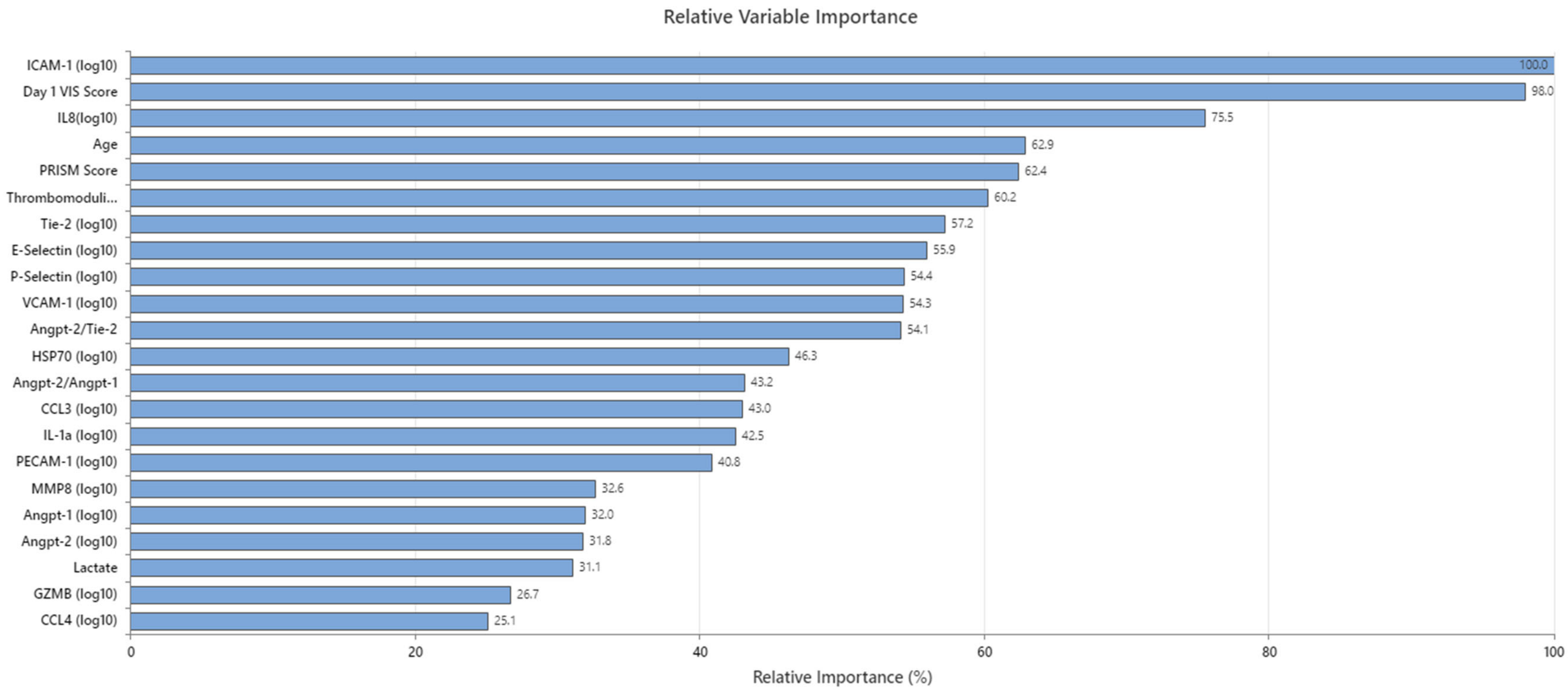

Variable importance measures model improvement when splits are made on a predictor. Relative importance is defined as % improvement with respect to the top predictor.

Relative variable importance of 22 variable TreeNet® PERSEVERence model to predict day 7 renal dysfunction

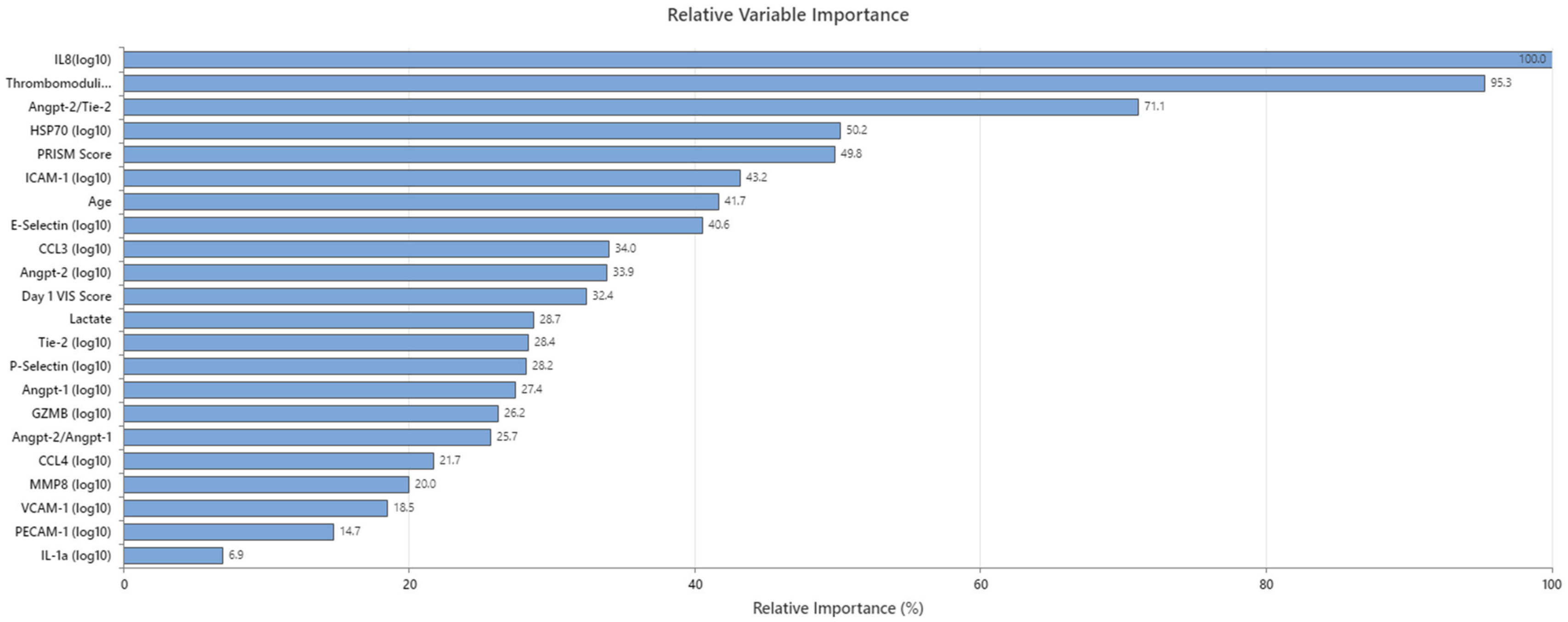

Variable importance measures model improvement when splits are made on a predictor. Relative importance is defined as % improvement with respect to the top predictor.

Relative variable importance of 22 variable TreeNet® PERSEVEREnce model to predict day 7 hepatic dysfunction

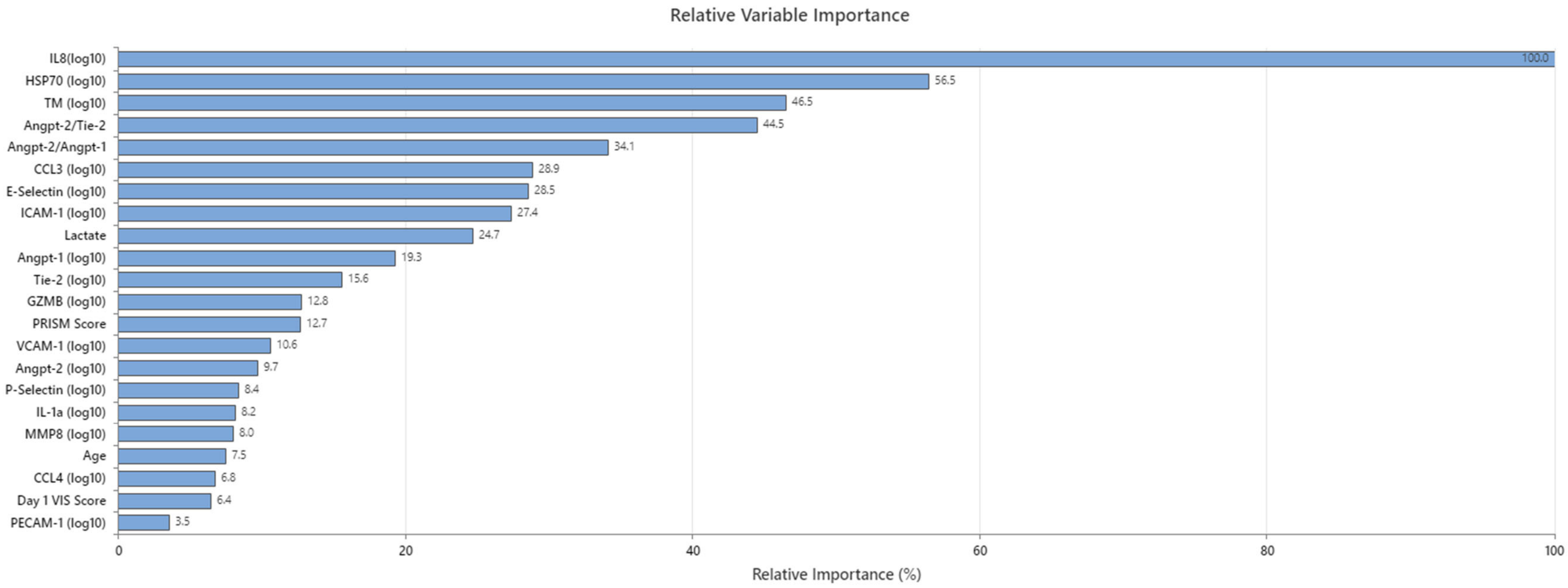

Variable importance measures model improvement when splits are made on a predictor. Relative importance is defined as % improvement with respect to the top predictor.

Relative variable importance of 22 variable TreeNet® PERSEVEREnce model to predict day 7 hematologic dysfunction.

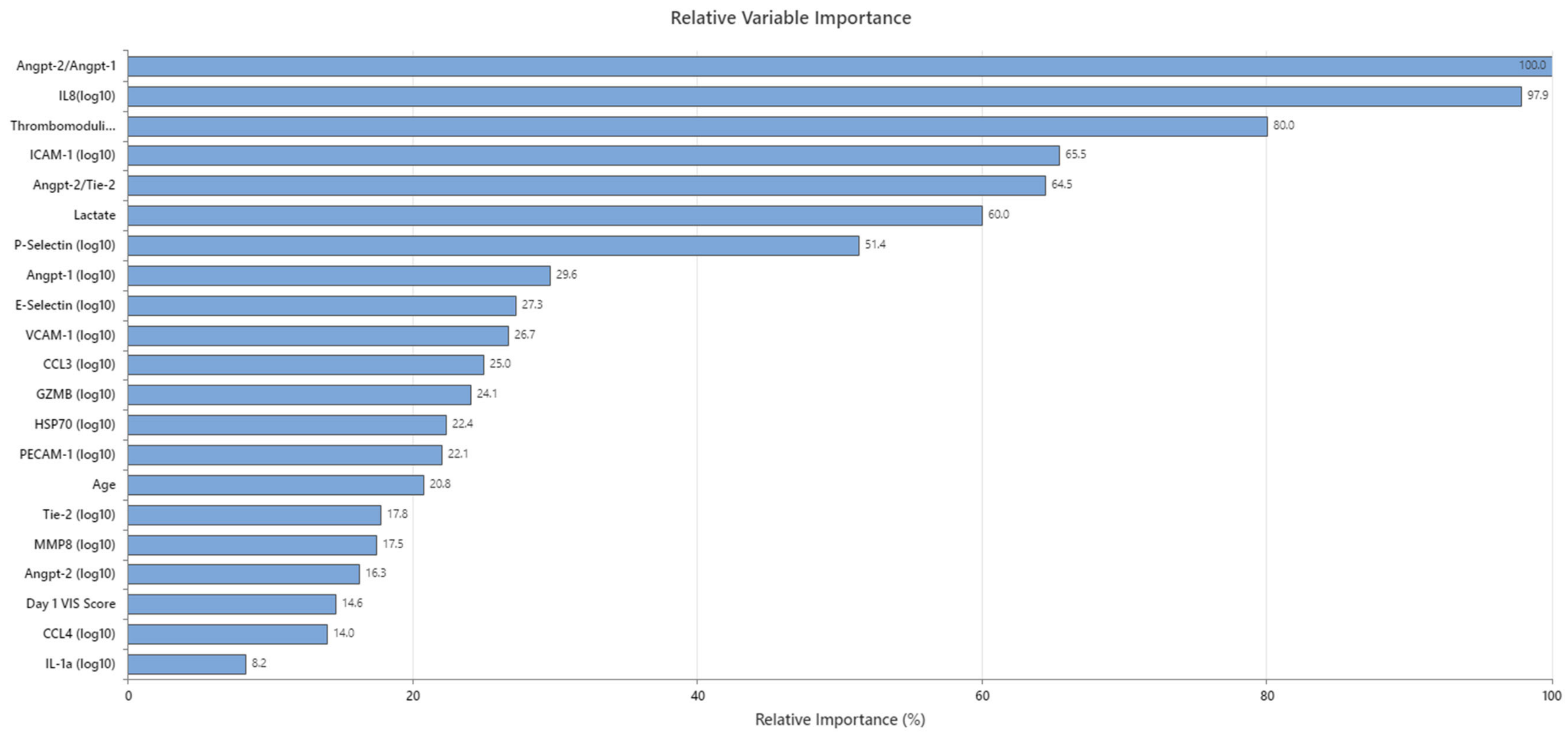

Variable importance measures model improvement when splits are made on a predictor. Relative importance is defined as % improvement with respect to the top predictor.

Relative variable importance of 22 variable TreeNet® PERSEVEREnce model to predict day 7 neurologic dysfunction.

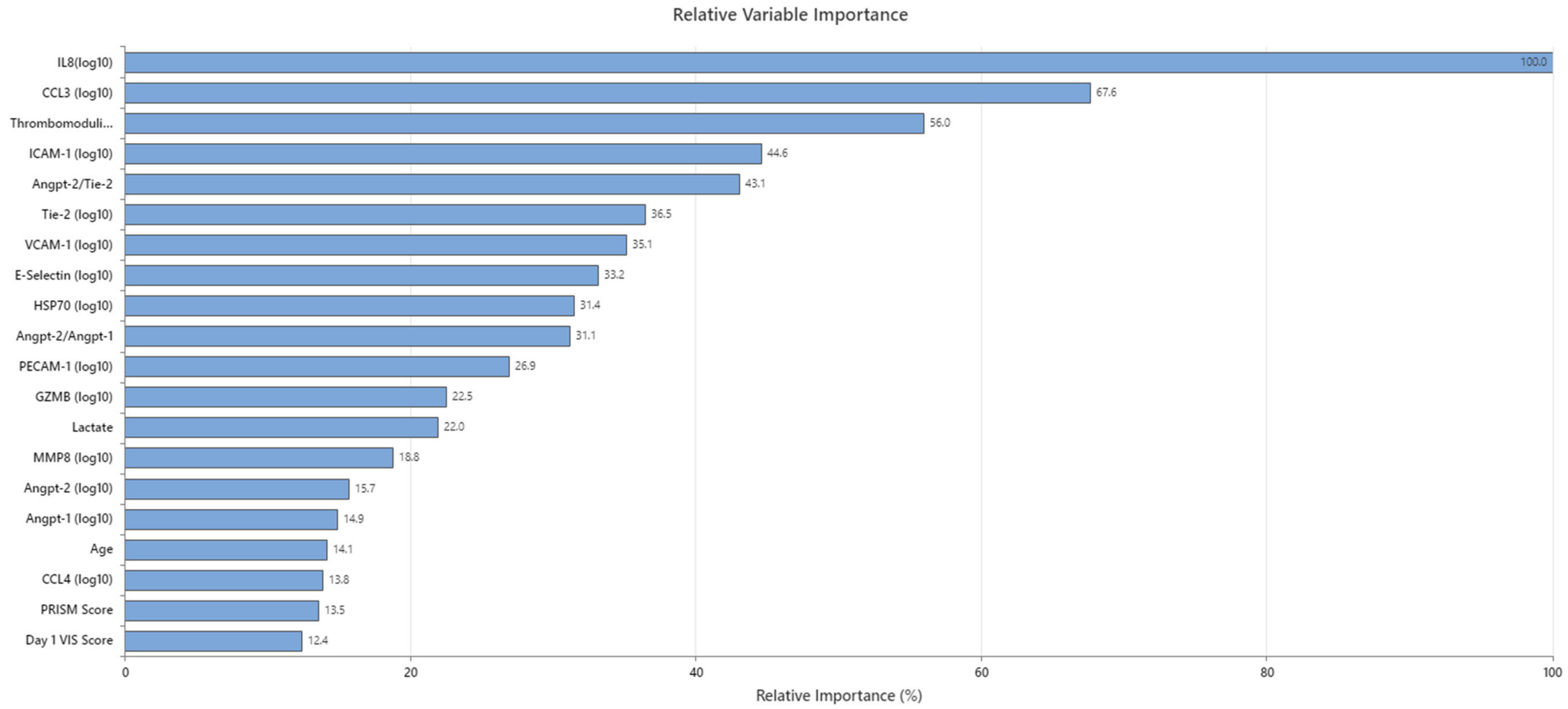

Variable importance measures model improvement when splits are made on a predictor. Relative importance is defined as % improvement with respect to the top predictor.
