# Supplementary material for: Integrated PERSEVERE and endothelial biomarker risk model predicts death and persistent MODS in pediatric septic shock: a secondary analysis of a prospective observational study
Source: Crit Care. 2022 Jul 11;26:210. doi: 10.1186/s13054-022-04070-5 (PMC9275255; doi:10.1186/s13054-022-04070-5)
Supplement: Supplementary file 6 — Additional file 6. Figure shows top two-way interaction between variables in PERSEVEREnce model to estimate risk of death or day 7 MODS. Table shows top two-way interactions in the organ-specific PERSEVEREnce models. [file 13054_2022_4070_MOESM6_ESM.pdf]

Additional file 6:

Supplemental Figure 4: Surface and contour plots of top two-way interacting variables and risk of death or day 7 MODS

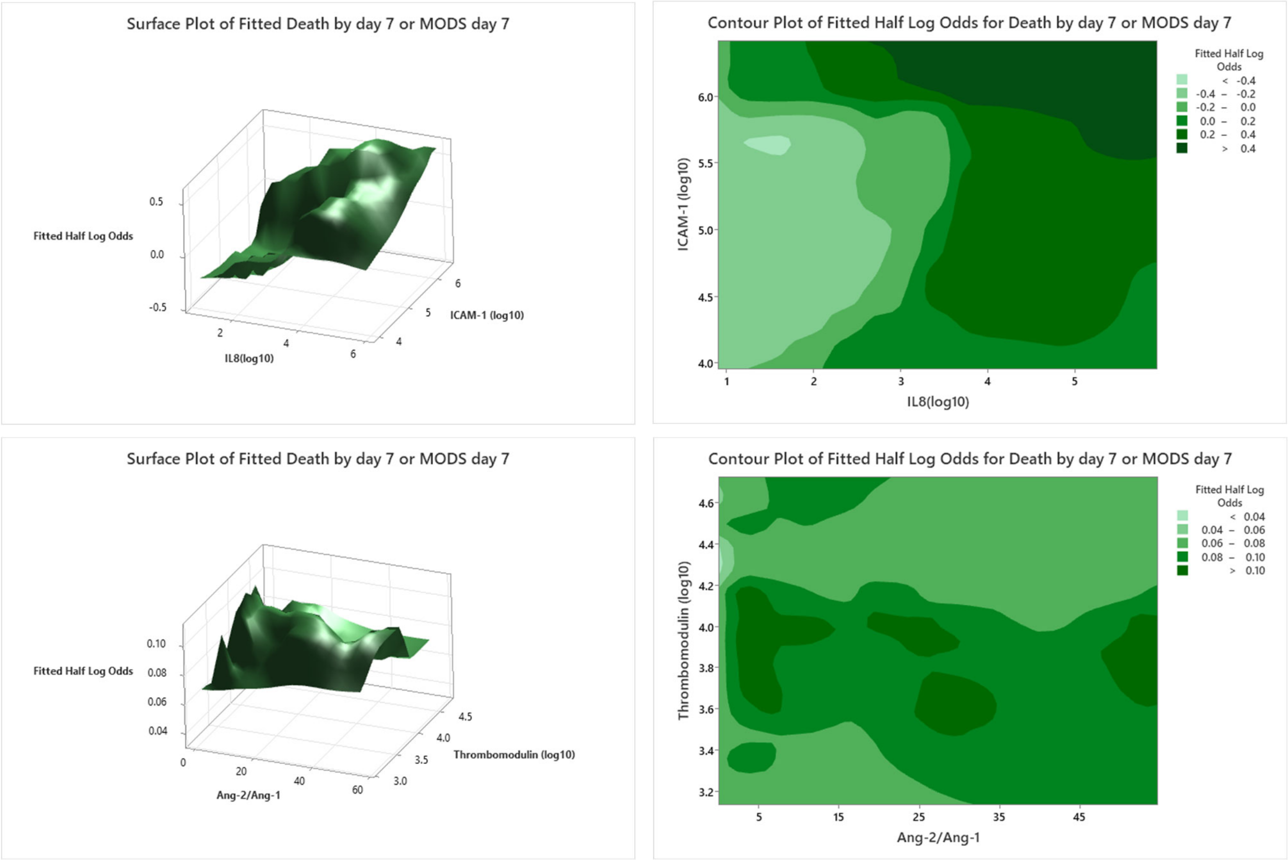

Table: Top 2-way interactions between predictors in 22 variable TreeNet® organ-specific PERSEVEREnce risk models.

Outcome	% of total squared error **	% of squared error ***	Predictor 1	Predictor 2
Day 7 CVS Dysfunction	7.5	21.1	IL-8 (Log10)	Angpt-2/Tie-2
	4.1	20.1	CCL3 (log10)	Angpt-2/Angpt-1
Day 7 Resp Dysfunction	2.4	12.1	HSP70 (log10)	IL-8 (Log10)
	1.8	10.3	VCAM-1	Angpt-2/Tie-2
Day 7 Renal Dysfunction	11.3	17.5	IL-8 (Log10)	Thrombomodulin (log10)
	6.5	12.5	IL-8 (Log10)	Angpt-2/Tie-2
Day 7 Hepatic Dysfunction	8.5	11.6	IL-8 (Log10)	Angpt-2/Angpt-1
	7.8	10.6	IL-8 (Log10)	Thrombomodulin (log10)
Day 7 Hematologic Dysfunction	8.3	13.7	IL-8 (Log10)	Angpt-2/Angpt-1
	5.6	13.3	Thrombomodulin (log10)	Angpt-2/Angpt-1
Day 7 Neurologic Dysfunction	26.9	38.8	IL-8 (Log10)	Thrombomodulin (log10)
	6.6	18.6	CCL3 (log10)	Angpt-2/Tie-2

** Percent of total variation in model that can be attributed to the two predictors including main effects and 2-way interaction.

*** Percent of variation in main and interaction effects of 2 predictor variables that can be attributed to the 2-way interaction effect.
