# Supplementary material for: Integrated PERSEVERE and endothelial biomarker risk model predicts death and persistent MODS in pediatric septic shock: a secondary analysis of a prospective observational study
Source: Crit Care. 2022 Jul 11;26:210. doi: 10.1186/s13054-022-04070-5 (PMC9275255; doi:10.1186/s13054-022-04070-5)
Supplement: Supplementary file 7 — Additional file 7. AUROC (Figure) and test characteristics (Table) for simplified PERSEVEREnce model to estimate risk of death or day 7 MODS. [file 13054_2022_4070_MOESM7_ESM.pdf]

Additional File 7:
Supplemental Figure 5: AUROC of the simplified 6 variable PERSEVEREnce risk model to predict death or day 7 MODS in children with septic shock.

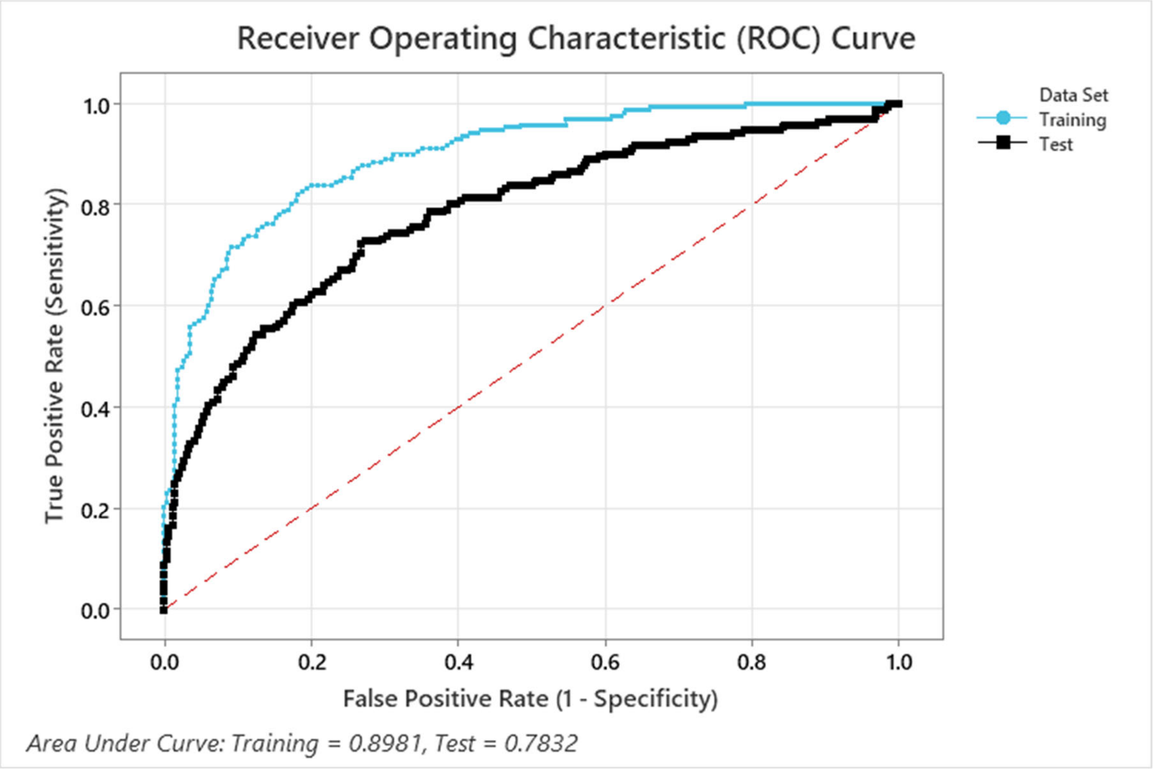

Table. Test characteristics of the 6 variable PERSEVEREnce model to estimate risk of death or day 7 MODS in children with septic shock.

	Training Set	Test Set
AUROC	0.89 (0.87-0.93)	0.78 (0.74-0.83)
Weighted misclassification rate	0.18	0.27
True positive, n	145	126
False negative, n	28	47
False positive, n	65	88
True negative, n	264	288
Sensitivity %	83.8 (77.2, 88.8)	72.8 (65.4, 79.1)
Specificity %	80.2 (75.4, 84.3)	76.5 (71.9, 80.7)
Positive predictive value %	69.1 (62.2, 75.1)	58.8 (51.9, 65.4)
Negative predictive value %	90.4 (86.3, 93.4)	85.9 (81.6, 89.4)
Positive likelihood ratio	4.2 (3.4, 5.3)	3.1 (2.5, 3.8)
Negative likelihood ratio	0.1 (0.1, 0.2)	0.2 (0.1, 0.3)
