# Supplementary material for: Integrated PERSEVERE and endothelial biomarker risk model predicts death and persistent MODS in pediatric septic shock: a secondary analysis of a prospective observational study
Source: Crit Care. 2022 Jul 11;26:210. doi: 10.1186/s13054-022-04070-5 (PMC9275255; doi:10.1186/s13054-022-04070-5)
Supplement: Supplementary file 8 — Additional file 8. Test characteristics of 6-variable organ-specific PERSEVEREnce risk models. [file 13054_2022_4070_MOESM8_ESM.pdf]

Additional file 8: Performance of 6 variable organ-specific PERSEVEREnce risk models.

		Training Set	Test Set
Day 7 Cardiovascular Dysfunction			
	AUROC	0.87 (0.84, 0.91)	0.77 (0.72, 0.83)
	Weighted misclassification rate	0.22	0.29
	True positive, n	108	97
	False negative, n	14	25
	False positive, n	124	139
	True negative, n	256	241
	Sensitivity %	88.5 (81.1, 93.3)	79.5 (71.1, 86.1)
	Specificity %	67.3 (62.3, 71.1)	63.4 (58.3, 68.2)
	Positive predictive value %	46.5 (40.1, 53.1)	41.1 (34.8, 47.6)
	Negative predictive value %	94.8 (91.3, 97.1)	90.6 (86.2, 93.7)
Day 7 Respiratory Dysfunction			
	AUROC	0.84 (0.91, 0.87)	0.66 (0.61, 0.71)
	Weighted misclassification rate	0.24	0.38
	True positive, n	149	120
	False negative, n	44	73
	False positive, n	80	120
	True negative, n	229	189
	Sensitivity %	77.2 (70.5, 82.7)	62.1 (54.9, 68.9)
	Specificity %	74.1 (68.7, 78.8)	61.1 (55.4, 66.5)
	Positive predictive value %	65.0 (58.4, 71.1)	50.0 (43.5, 56.4)
	Negative predictive value %	83.8 (78.8, 87.9)	72.1 (66.3, 77.3)
Day 7 Renal Dysfunction			
	AUROC	0.90 (0.87, 0.93)	0.79 (0.75, 0.79)
	Weighted misclassification rate	0.19	0.27
	True positive, n	120	102
	False negative, n	23	41
	False positive, n	76	87
	True negative, n	283	272
	Sensitivity %	83.9 (76.6, 89.3)	71.3 (63.0, 78.4)
	Specificity %	78.8 (74.1, 82.8)	75.7 (70.9, 80.0)
	Positive predictive value %	61.2 (54.0, 68.0)	53.9 (46.5, 61.1)
	Negative predictive value %	92.4 (88.7, 95.1)	86.9 (82.5, 90.3)

		Training Set	Test Set
Day 7 Hepatic Dysfunction			
	AUROC	0.97 (0.96, 0.98)	0.88 (0.85, 0.89)
	Weighted misclassification rate	0.08	0.20
	True positive, n	80	64
	False negative, n	3	19
	False positive, n	55	64
	True negative, n	364	355
	Sensitivity %	96.3 (89.1, 99.1)	77.1 (66.3, 85.3)
	Specificity %	86.8 (83.1, 89.8)	84.7 (80.8, 88.0)
	Positive predictive value %	59.2 (50.4, 67.5)	50.0 (41.0, 58.9)
	Negative predictive value %	99.2 (97.4, 99.7)	94.9 (92.1, 96.8)
Day 7 Hematologic Dysfunction			
	AUROC	0.93 (0.91, 0.96)	0.82 (0.77, 0.86)
	Weighted misclassification rate	0.14	0.25
	True positive, n	95	75
	False negative, n	12	32
	False positive, n	66	81
	True negative, n	329	314
	Sensitivity %	88.7 (80.8, 93.8)	70.1 (60.3, 78.3)
	Specificity %	83.3 (79.1, 86.7)	79.4 (75.1, 83.2)
	Positive predictive value %	59.0 (50.9, 66.6)	48.1 (40.1, 56.1)
	Negative predictive value %	96.4 (93.7, 98.1)	90.7 (87.1, 93.5)
Day 7 Neurologic Dysfunction			
	AUROC	0.92 (0.89, 0.95)	0.81 (0.74, 0.88)
	Weighted misclassification rate	0.13	0.22
	True positive, n	38	35
	False negative, n	3	6
	False positive, n	90	134
	True negative, n	371	327
	Sensitivity %	92.7 (78.9, 98.1)	85.4 (70.1, 93.9)
	Specificity %	80.4 (76.4, 83.9)	70.9 (66.5, 75.0)
	Positive predictive value %	29.7 (22.1, 38.5)	20.7 (15.0, 27.7)
	Negative predictive value %	99.1, 97.4, 99.7)	98.1 (95.9, 99.2)
