# Supplementary material for: Integrated PERSEVERE and endothelial biomarker risk model predicts death and persistent MODS in pediatric septic shock: a secondary analysis of a prospective observational study
Source: Crit Care. 2022 Jul 11;26:210. doi: 10.1186/s13054-022-04070-5 (PMC9275255; doi:10.1186/s13054-022-04070-5)
Supplement: Supplementary file 9 — Additional file 9. Top two-way interactions in the simplified organ-specific PERSEVEREnce risk models. [file 13054_2022_4070_MOESM9_ESM.pdf]

Additional file 9: Top 2-way interactions between predictors in 6 variable TreeNet® organ-specific PERSEVEREnce risk models.

Outcome	% of total squared error **	% of squared error ***	Predictor 1	Predictor 2
Day 7 CVS Dysfunction	12.3	26.5	IL-8 (Log10)	Angpt-2/Tie-2
	12.3	13.9	ICAM-1	Angpt-2/Tie-2
Day 7 Resp Dysfunction	8.3	19.6	HSP70 (log10)	ICAM-1 (log10)
	8.2	12.2	VCAM-1	Angpt-2/Tie-2
Day 7 Renal Dysfunction	11.6	16.3	IL-8 (Log10)	Thrombomodulin (log10)
	9.7	15.6	IL-8 (Log10)	Angpt-2/Tie-2
Day 7 Hepatic Dysfunction	10.4	13.9	IL-8 (Log10)	IL-8 (Log10)
	10.3	12.4	HSP70 (log10)	Thrombomodulin (log10)
Day 7 Hematologic Dysfunction	12.3	18.5	IL-8 (Log10)	Angpt-2/Angpt-1
	6.4	11.1	Thrombomodulin (log10)	Angpt-2/Angpt-1
Day 7 Neurologic Dysfunction	37.8	42.2	IL-8 (Log10)	Thrombomodulin (log10)
	11.9	14.1	IL-8 (Log10)	Angpt-2/Tie-2

** Percent of total variation in model that can be attributed to the two predictors including main effects and 2-way interaction.

*** Percent of variation in main and interaction effects of 2 predictor variables that can be attributed to the 2-way interaction effect.
