# Supplementary figures and images for: Integrated PERSEVERE and endothelial biomarker risk model predicts death and persistent MODS in pediatric septic shock: a secondary analysis of a prospective observational study
Source: Crit Care. 2022 Jul 11;26:210. doi: 10.1186/s13054-022-04070-5 (PMC9275255; doi:10.1186/s13054-022-04070-5)

Additional File 10: Supplemental Figure 6

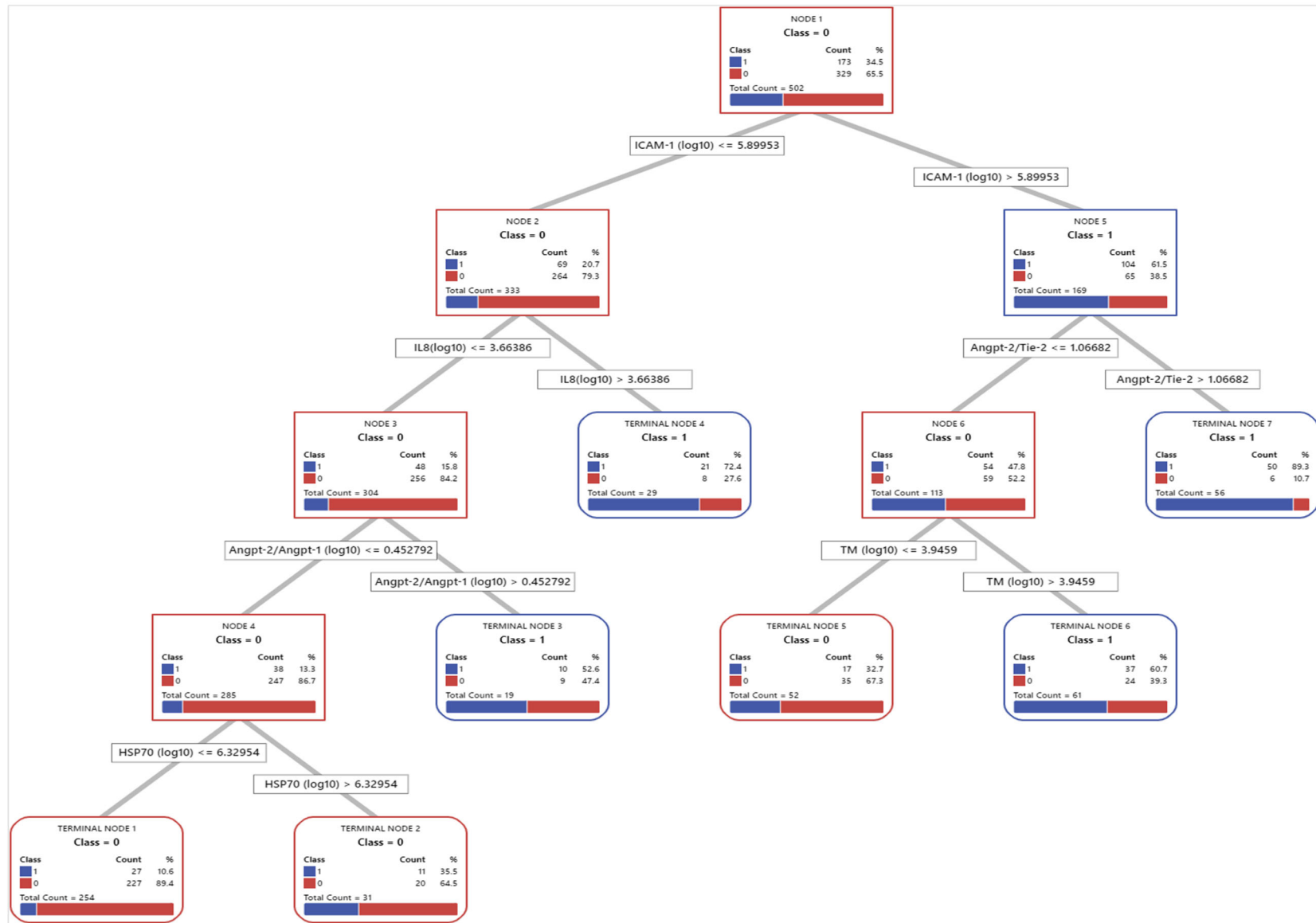

Supplement: Supplementary file 10 — Additional file 10. Seven terminal node CART tree model to estimate risk of death or day 7 MODS in children with septic shock. [file 13054_2022_4070_MOESM10_ESM.pdf]
